# Hepatomas are exquisitely sensitive to pharmacologic ascorbate (P-AscH^-^)

**DOI:** 10.7150/thno.35378

**Published:** 2019-10-18

**Authors:** Xuan Zhang, Tiefu Liu, Zehuan Li, Yanling Feng, Christopher Corpe, Shanshan Liu, Jingpu Zhang, Xiaomeng He, Feng Liu, Li Xu, Longqiang Shen, Shun Li, Qianlin Xia, Xiuhua Peng, Xiaohui Zhou, Weiping Chen, Xiaoyan Zhang, Jianqing Xu, Jin Wang

**Affiliations:** 1 Shanghai Public Health Clinical Center, Fudan University, 2901 Caolang Road, Jinshan District, Shanghai 201508, China;; 2 Department of General Surgery, Zhongshan Hospital, Fudan University, 200032, Shanghai, China;; 3 King's College London, London, Nutritional Science Department, 150 Stamford street, waterloo, London, SE19NH, United Kingdom;; 4 Department of Laboratory Medicine, Shanghai Jiao Tong University Affiliated Sixth People's Hospital, Shanghai, China;; 5 Genomics Core, National Institute of Diabetes and Digestive and Kidney Diseases, National Institutes of Health, Bethesda, MD 20892, USA.

**Keywords:** Ascorbate, vitamin C, cancer, hepatoma, ROS, mitochondrial respiration.

## Abstract

**Rationale:** Ascorbate is an essential micronutrient known for redox functions at normal physiologic concentrations. In recent decades, pharmacological ascorbate has been found to selectively kill tumour cells. However, the dosing frequency of pharmacologic ascorbate in humans has not yet been defined.

**Methods:** We determined that among five hepatic cell lines, Huh-7 cells were the most sensitive to ascorbate. The effects of high-dose ascorbate on hepatoma were therefore assessed using Huh-7 cells and xenograft tumour mouse model.

**Results:** In Huh-7 cells, ascorbate induced a significant increase in the percentage of cells in the *G0/G1* phase, apoptosis and intracellular levels of ROS. High doses of ascorbate (4.0 pmol cell^-1^), but not low doses of ascorbate (1.0 pmol cell^-1^), also served as a pro-drug that killed hepatoma cells by altering mitochondrial respiration. Furthermore, in a Huh-7 cell xenograft tumour mouse model, intraperitoneal injection of ascorbate (4.0 g/kg/3 days) but not a lower dose of ascorbate (2.0 g/kg/3 days) significantly inhibited tumour growth. Gene array analysis of HCC tumour tissue from xenograft mice given IP ascorbate (4.0 g/kg/3 days) identified changes in the transcript levels of 192 genes/ncRNAs involved in insulin receptor signalling, metabolism and mitochondrial respiration. Consistent with the array data, gene expression levels of *AGER, DGKK, ASB2, TCP10L2, Lnc-ALCAM-3*, and* Lnc-TGFBR2-1* were increased 2.05-11.35 fold in HCC tumour tissue samples from mice treated with high-dose ascorbate, and IHC staining analysis also verified that AGER/RAGE and DGKK proteins were up-regulated, which implied that *AGER/RAGE* and *DGKK* activation might be related to oxidative stress, leading to hepatoma cell death.

**Conclusions:** Our studies identified multiple mechanisms are responsible for the anti-tumour activity of ascorbate and suggest high doses of ascorbate with less frequency will act as a novel therapeutic agent for liver cancer *in vivo*.

## Introduction

Vitamin C, also known as ascorbic acid (L-ascorbic acid, Asc), is an essential micronutrient for humans acting as a cofactor for various biosynthetic enzymes, especially those involved in scavenging reactive oxygen species (ROS) 1, 2. Asc is also a weak acid (pKa of 4.1 - 4.2) and at physiologic pH (7.4) it exists predominantly in the anionic form (AscH^-^). Over 50 years ago Cameron and Pauling suggested Asc (or AscH^-^) could be a potential drug for the treatment of cancer 3, 4. However, its efficacy was refuted by subsequent double-blind studies that failed to show improvements in the survival of patients receiving oral vitamin C compared to placebo 5, 6. Several more recent studies have shown IV ascorbate was an effective anticancer treatment for solid tumours when compared with orally administered ascorbate 1, 7. Preclinical and clinical studies in cholangiocarcinoma (CC) 8, colon cancer 9, 10, glioblastoma multiforme (GBM) 11, melanoma 12, non-small cell lung cancer (NSCLC) 11, ovarian cancer 13, pancreatic cancer 14, and sarcoma 15 have revealed pharmacologic levels of ascorbate achievable by IV but not oral administration selectively kills cancer cells but not normal cells. Pharmacologic ascorbate (P-AscH^-^) *in vivo* (> 1.0 mM) could be reached in patients by IV injection (at an average dose of 0.5 g/kg) to kill cancer cells, without side effects 1, 10, 16. Thus, identification of tumour types that are exquisitely sensitive to high doses of ascorbate in preclinical models can advance clinical testing.

The efficacy of vitamin C treatment could not be judged from clinical trials if using only oral dosing, and only high intravenous doses of vitamin C produced high plasma concentrations that might have antitumor activity, moreover pharmacokinetic data at high intravenous doses of vitamin C in cancer patients are sparse 17. Dr. Levine noticed when 1.25 g of vitamin C was given intravenously; plasma concentrations were significantly higher than when the vitamin was given orally 18. At extracellular concentrations > 1.0 mM vitamin C was toxic to some cancer cells, possibly because high concentrations of vitamin C act as a pro-drug for hydrogen peroxide formation in plasma 18, 19. In addition, the elucidation of mechanisms of cancer-selective cell death induced by ascorbate may also provide insight into liver cancer therapy. Rouleau verified that the extracellular formation of H_2_O_2_ by high doses of ascorbate was a prerequisite for cancer cell death via increased cytosolic calcium, which in turn promoted mitochondrial calcium uptake and oxidative metabolism in cancer cells 20. Current clinical evidence on the therapeutic effect of high-dose IV ascorbate is ambiguous. We proposed a hypothesis that extracellular H_2_O_2_ formation is a key mediator of cell death by pharmacologic ascorbate, and that H_2_O_2_ can cause death by multiple, distinct mechanisms in the same cell type. Only high doses of ascorbate have been described to possess anticancer effects, but the potential mechanisms of action are unclear.

Hepatocellular carcinoma (HCC) is the third most common cause of cancer-related mortality worldwide and is usually diagnosed at a late stage 21, 22. Although alternative strategies with sorafenib, lenvatinib and regorafenib might improve survival in patients with advanced HCC, the only potentially curative treatment for HCC is tumour resection. Moreover, only approximately 15% of HCC patients are amenable to operative treatment, and the chance that treatment for HCC will be curative remains low 23, 24. HCC is therefore a clinical problem in urgent need of novel and effective anticancer approaches. Because there is an abundance of iron in liver and pharmacologic ascorbate kills various cancer cells by producing extracellular hydrogen peroxide via Fenton chemistry 7, 25-27 involving redox-active labile iron, we hypothesized that hepatoma cells might be more sensitive to pharmacologic ascorbate. However, a difficulty of using pharmacologic ascorbate is dosing frequency intervals which to date have not been described 28.

In this study we first investigated ascorbate-induced cytotoxicity towards Huh-7, HCCLM9, MHCC97L and LO2 cells and demonstrated that Huh-7 cells were the cells most sensitive to ascorbate and hydrogen peroxide via mitochondrial dysfunction. We further assessed the effects of P-AscH^-^ on mice with HCC *in vivo* and found the tumour growth was significantly reduced after IP injection of ascorbate at 4.0 g/kg/3 days compared to the tumour growth in the PBS control group. Gene array analysis identified the upregulation of* AGER, DGKK, ASB2, TCP10L2, Lnc-ALCAM-3*, and *Lnc-TGFBR2-1* which were validated by qPCR. Peroxide induced mitochondrial dysfunction in HCC was also detected leading to cell death. Thus, our dose-response studies of ascorbate in cells, a xenograft tumour mouse model and the two case studies using high dose ascorbate treated HCC patients identified a big theoretical advantage of ascorbate in cancer treatment via multiple mechanisms.

## Materials and Methods

**Cell lines and cell culture.** Human hepatic cells (LO2, HepG2, MHCC97L, and HCCLM9 cells) were cultured in RPMI-1640 medium, and Huh-7 cells were maintained in DMEM (Dulbecco's Modified Eagle Medium, Thermo Fisher) supplemented with 10% foetal bovine serum (FBS, Biological Industries) and 1% penicillin and streptomycin and cultured in a humidified atmosphere of 37℃ with 5% CO_2_. Human LO2 and Huh-7 cells were purchased from the cell bank of the Shanghai Institute of Biochemistry and Cell Biology (Shanghai, China). MHCC97L and HCCLM9 cells were derived from the same host cell line MHCC97L as described previously 29.

**Cell viability and soft-agar assay.** Human hepatic cells were seeded on a 96-well plate (1.0 x 10^4^ cells/well). The ascorbate dose gradient was set as 4.0 mM at the highest, with double dilution to 15.63 μM. Cells were incubated with ascorbate for 24 hrs, followed by removal of the ascorbate from the cultured medium. Then, 10 μL of 5 mg/mL MTT (Thiazolyl Blue Tetrazolium Bromide, Sigma) was added to each well for 4 hrs, and 150 μL DMSO was added to each well to dissolve the formazan. The relative absorbance (λ = 570 nm) for each well was detected in an ELx808™ Absorbance Microplate Reader (BioTek, Winooski, VT, USA). The LD_50_ was determined from the plots of the percentage viability vs. the dose of compound added as described previously 30. For the soft-agar assay, the five hepatic cells (1.0 × 10^3^) were exposed to ascorbate for 1 hr then washed and re-suspended in RPMI 1640 culture Medium (ThermoFisher Scientific, Waltham, MA) supplemented with 10% FBS (ThermoFisher Scientific) for 0.3% ultra-pure agarose (Sigma-Aldrich, St. Louis, MO). The suspension was then poured over 3 mL of pre-solidified 0.6% agar base (Sigma-Aldrich) in 60 mm^2^ dishes, and the plates incubated at 37°C with 5% CO_2_ atmosphere saturation. Plates were incubated for 14-21 days at 37°C, 5% CO_2_, and the growth medium on top of the agarose layer replaced each week. After the growth period, cells were fixed with 70% ethanol and stained with Coomassie Blue. All the colonies were counted in the fields (n = 9) of view which were photographed using an inverted phase microscope (Olympus CKX4SF; Tokyo, Japan) at 40× magnification. The plating efficiency and surviving fraction were determined by the clonogenic survival fraction vs. dose of ascorbate.

**Cell cycle and apoptosis analysis by flow cytometry.** Huh-7 cells (1 x 10^5^ cells / 6 cm plate) were exposed to ascorbate 0-10 pmol cell^-1^ for 24 hrs. The treated cells were harvested and fixed overnight with 70% ethanol at 4°C. The fixed cells were washed twice with cold PBS containing 0.1% Triton, suspended in 20 µg/µL propidium iodide (PI), 200 μg/mL RNase and 0.1% Triton X-100, and incubated for 20 min in the dark at 37°C. Cell cycle distribution was determined by fluorescence-activated cell sorting analysis of PI-stained ethanol-fixed cells using a Guava EasyCyte (Guava Technologies, Hayward, CA). For apoptosis analysis, the cells were measured using the FITC Annexin V Apoptosis Detection Kit with PI (Cat^#^: 640914, BioLegend Inc., San Diego, CA, USA) according to the manufacturer's instructions. As mentioned above, Huh-7 cells (1 x 10^5^ cells / 6 cm plate) were exposed to ascorbate and H_2_O_2_ for 24 hrs. Adherent Huh-7 cells were collected and washed with BioLegend's Cell Staining Buffer, then suspended in a mixture staining buffer comprised of 45 μL Annexin V binding buffer, 2.5 μL FITC-Annexing V, and 2.5 μL PI for 15 min in the dark at 37°C. Apoptosis was measured by a FACSCalibur flow cytometer (BD Biosciences, San Jose, CA, USA). Data were analysed by FlowJo™ version 7.6.3 software (TreeStar, Ashland, OR, USA) and R software.

**Mitochondrial respiration and glycolysis stress test.** The dynamics of mitochondrial oxidative phosphorylation or glycolysis were respectively assessed by comparing oxygen consumption rate (OCR) and extracellular acidification rate (ECAR) using a Cell Mito Stress Test Kit (Cat#: 103015-100, Seahorse Bioscience Santa Clara, California, USA) or Glycolysis Stress Test Kit (Cat#: 103020-100, Seahorse Bioscience Santa Clara, California, USA) on an XFe-24 Extracellular Flux Analyzer (Seahorse Bioscience) as described previously 31. In brief, Huh-7 cells (5.0 x 10^4^ cells / well XFe-24 cell culture microplate) were grown in DMEM supplemented with 10% foetal bovine serum and treated for 1 h with or without ascorbate (1.0, 4.0, 8.0, or 10 pmol cell^-1^). Cells were then washed twice with basal assay medium and pre-incubated for 1 h in a CO_2_-free incubator in 500 μL/well of assay medium containing 10 mM glucose, 1.0 mM pyruvate and 2.0 mM L-glutamine. An XFe-24 Sensor Cartridge containing 1.0 mL/well of Seahorse XFe Calibrant was preincubated overnight at 37°C in a non-CO_2_ incubator. For Mito Stress Test, ten-fold concentrated compounds in the kit of oligomycin (Complex V inhibitor), carbonyl cyanide-4 (trifluoromethoxy) phenylhydrazone (FCCP, mitochondrial uncoupler), and a mixture of rotenone (complex I inhibitor) and antimycin A (complex III inhibitor) were loaded into the XFe-24 Sensor Cartridge to produce final concentrations of 1.0 μM, 1.0 μM, 0.5 μM and 0.5 μM, respectively. For the Glycolysis stress test, ten-fold concentrations of compounds from the kit containing glucose (fuel for glycolysis), oligomycin (Complex V inhibitor) and 2-Deoxy-D-Glucose (2-DG, competitive inhibitor of hexokinase) were loaded into the matched cartridge to produce final concentrations of 10 mM, 1.0 μM and 50 mM, respectively. After a 30-min calibration of the XF sensor with the preincubated sensor cartridge, the cell plates were separately loaded into the analyser, and mitochondrial respiratory and glycolysis parameters were analysed under basal conditions followed by the sequential injection of the complex inhibitors oligomycin, FCCP. For the Mito stress test a mixture of rotenone and antimycin A was added, and for the Glycolysis stress test, glucose, oligomycin and 2-DG were added. Moreover, in Mito stress test, ATP production was evaluated by the difference between the basal OCR and the OCR after oligomycin injection; spare respiratory capacity (SRC) was determined as the difference between maximal and basal OCRs; whereas, in Glycolysis Stress Test, glycolysis was evaluated as the difference in ECAR before oligomycin injection and glucose injection; non-glycolytic acidification was determined as the last ECAR prior to glucose injection. Data were analysed using Seahorse Wave 2.4 Software (Seahorse Bioscience) and normalized with cell number or proteins (Pierce BCA Protein Assay Kit,Thermo Fisher Scientific) loaded in each well. Quadruplicates of each cell treatment were analysed.

**Extracellular ascorbate oxidation analysis.** The five hepatic cells (5.0 x 10^4^ cells / well XFe-24 cell culture microplate) were grown in DMEM supplemented with 10% foetal bovine serum overnight. The rate of oxygen consumption of the five hepatic cell lines upon addition of ascorbate (3.0 mM) to the complete culture mediums was determined using a XFe-24 Extracellular Flux Analyzer (Seahorse Bioscience). The OCR represents the rate of H_2_O_2_ production. The accumulation of H_2_O_2_ is determined through the addition of catalase (250 units mL^-1^) (bovine liver, Sigma C-1345) as described previously 32.

**Intracellular ATP analysis.** Intracellular ATP levels in Huh-7 cell were measured using the ATP Assay Kit (Beyotime Biotechnology, Shanghai, China). Huh-7 cells (1 x 10^5^ cells / well) were exposed to ascorbate for 1 hr in six-well plate and washed by PBS. Then, 200 μL of lysis buffer was added to lyse the cells. After centrifugation at 12,000 for 5 min, 100 μL of ATP assay reagent was added to initiate the luminescence reaction. After 10 min, 20 μL of the supernatants was added to the ATP assay reagent and luminescence was measured on a Lumat LB 9507 tube luminometer (Berthold Technologies GmbH & Co. KG, Bad Wildbad, Germany). ATP standard curves with concentrations between 0 and 1000 μM were generated for each experiment. The ATP concentration was determined from the corresponding standard curve and converted to an intracellular concentration using the cell number/well as counted on a hemocytometer and normalized using protein concentration/well (Pierce BCA Protein Assay Kit, Thermo Fisher Scientific).

**Intracellular reactive oxygen species (ROS) level detection.** Dihydroethidium (DHE) was used to measure the intracellular level of ROS. Huh-7 cells (1 x 10^5^ cells / 6 cm plate) were incubated with concentrations of ascorbate (0 - 10 pmol Cell^-1^) for 1 hr, washed and then incubated with ascorbate-free medium for 5 hrs for ROS analysis. After treatment, the cells were washed twice with PBS and incubated with 2.0 μM DHE at 37℃ in the dark for 30 min. Stained cells were washed, resuspended in PBS, and analysed using a FACStar flow cytometer and FlowJo analytical software.

**Measurement of catalase activity.** Catalase activity was measured in Huh-7, HepG2, HCCLM9, MHCC97L and LO2 cell lysates using a spectrophotometric-based assay kit (Beyotime Biotechnology, Shanghai, China). Briefly, cells (1.0 × 10^6^) were harvested in 5.0 mL PBS. Cells were counted with the hemacytometer, so a well-defined number of cells was used in the assay. After cell lysis and centrifugation at 12,000 rpm for 5 min, the supernatants and catalase assay buffer were added to yield H_2_O_2_. The decomposition of H_2_O_2_ was measured at 520 nm in an ELx808™ Absorbance Microplate Reader (BioTek, Winooski, VT, USA).

**Glucose uptake assay.** Glucose uptake per cell was measured using the Glucose uptake cell-based assay kit (Cayman Chemical, Ann Arbor, MI, USA). 1 x 10^5^ cells / mL were grown in 2.0 mL culture medium in 6- well plates overnight, and were then treated with different ascorbate for 1 hr. After medium was removed, the cells were rinsed in PBS, and incubated at 37°C in 2 mL culture medium for 5 hrs. The cells were then incubated in triplicate with 20 µM 2-NBDG2-[N-(7-nitrobenz-2-oxa-1,3-diazol-4-yl) amino]-2-deoxy-D-glucose (2-NBDG) at 37^◦^C for 20 min in 5% CO_2_, and washed with FBS free DMEM medium with 4.5 g/L D-glucose for 5 min. Finally, the cells were washed twice with prechilled PBS and 100 μL of cell lysis buffer added. After centrifugation at 12,000 × g for 5 min at 4°C, fluorescence of aliquots from supernatants was measured by a fluorescence microplate assay 33. At the same time, a standard curve was generated by measuring the fluorescence of 2.5-50 μM 2-NBDG in lysis buffer.

**Xenograft tumour mouse model and bioluminescence imaging**. All mice were handled in strict accordance with good animal practice and institutional guidelines using an animal protocol approved by the Institutional Animal Care and Use Committee of the Shanghai Public Health Clinical Center. For the xenograft tumour model, we developed Huh-7 cells bearing lentivirus-luciferase, which was further modified to stably express the firefly luciferase gene by lentivirus transfection to facilitate the *in vivo* monitoring of tumour development. These cells were cultured to 80% confluence, harvested by trypsinization, washed twice by PBS, resuspended to a final concentration of 2 x 10^6^ cells / 100 μL in sterile PBS, and injected subcutaneously into the right flank of 6-week-old male nude mice. When the tumour volume reached 25 - 50 mm^3^, the nude mice with subcutaneous Huh-7 cell tumours were randomly divided into three groups: two treatment groups given IP injections of 200 μL and 400 μL of Vitamin C respectively (Cat^#^: H310211486; 5 mL: 1g; Shanghai Harvest Pharmaceutical Co. Ltd.) (2.0 g/kg or 4.0 g/kg ascorbate) and a control group given an equivalent volume of normal saline (PBS) (7 mice in each group). The weight of the mice was measured every two days.

**Bioluminescence imaging analysis**. Tumour growth was assessed by bioluminescence imaging. Before they were imaged, the mice in each group were anaesthetized with 3% sodium pentobarbital via intraperitoneal injection. Then, 50 mg/kg pentobarbital sodium was injected intraperitoneally into each mouse after anaesthesia; mice were then given an IP injection of 150 mg/kg D-luciferin, and the image was captured after 30 min. The anaesthetized mice were placed in the heated imaging platform of an IVIS-100/Spectrum optical imaging system (Xenogen / Caliper, Mountain View, CA). Signal intensity was quantified as the sum of all detected photons within the region of interest per second. Tumours were fixed with formaldehyde and histologically evaluated to verify the accuracy of the bioluminescence data. Acquired images were analysed by the Living Image 3.1 software (Xenogen/Caliper, Alameda, CA). Fluorescence contrast, defined as radiance, was quantified using identical size regions of interest. The mice were killed humanely after 6 weeks, and the tumour was harvested for RNA, protein and immunohistochemical (IHC) analysis.

**RNA extraction and purification, gene expression profiling and data analysis.** Total RNA was extracted from each group of cells using Trizol reagent following the manufacturer's instructions. RNA integrity and concentration were assessed using an Agilent Bioanalyzer 2100 (Agilent technologies, Santa Clara, CA, US) and Nano Drop ND-2000 (Nanodrop). For gene expression profiling, 1.0 μg RNA was amplified and labelled by the Low Input Quick Amp Labelling Kit, One-Color (Agilent Technologies, Santa Clara, CA, US) for one-colour processing, following the manufacturer's instructions. The method used T7 RNA Polymerase Blend, which simultaneously amplified the target material and incorporated Cy3-CTP. Labelled cRNA was purified by a RNeasy mini kit (QIAGEN, GmBH, Germany). Then, 600 ng Cy3-labelled cRNA was hybridized to the Agilent SurePrint G3 Human Gene Expression Microarray (8 x 60K) for 17 hrs, washed with the Gene Expression Wash Buffer Kit, and scanned by Agilent Microarray Scanner (Agilent Technologies, Santa Clara, CA, US), following the manufacturer's instructions. Data were extracted with Feature Extraction software 10.7 (Agilent Technologies, Santa Clara, CA, US) and analysed by R software. Gene Ontology (GO) analysis and KEGG (Kyoto Encyclopedia of Genes and Genomes) were performed to construct meaningful annotation of differentially expressed genes in HCC tumor tissue from mice treated with IP injection of ascorbate 34.

**Quantitative reverse transcription-polymerase chain reaction (qRT-PCR).** Five micrograms of RNA were used for the RT reaction cDNA synthesis step. The qPCR reactions were performed by the ABI StepOne Cycler. SYBR^®^ Premix Ex Taq^TM^ Ⅱ was purchased from TAKARA. The cycling procedure was as follows: 94°C for 30 s, 60°C for 30 s (40 cycles).* CDK4, CDK6, c-Myc, Casp3, AGER, DGKK, ASB2, TCP10L2, Lnc-ALCAM-3*, and *Lnc-TGFBR2-1* expression was assayed in xenograft tumour mice by qRT-PCR and the primer sequences were listed in Table S1. The individual Ct of the target gene was obtained from three different samples in each group, standardized by the Ct of the internal reference 18S. The fold change in transcriptional level relative to the control group was standardized by the ΔΔCt method.

**Immunohistochemical staining.** Immunohistochemical (IHC) staining for DGKK (Cat^#^: ab103681, Abcam) and AGER/RAGE (A-9) (Cat^#^: sc-365154, Santa Cruz Biotechnology, Inc.) was performed in 18 formalin-fixed, paraffin-embedded HCC mouse tumour tissue samples. The study was approved by the Shanghai Public Health Clinical Center institutional review boards. The DGKK and AGER staining results were independently evaluated by two expert pathologists (Feng Y and Li Z).

**Western blots.** Protein samples were lysed in RIPA buffer supplemented with protease inhibitors. Thirty micrograms of total protein were loaded per lane separated on a 10% sodium dodecyl sulfate-polyacrylamide gel by electrophoresis, and proteins transferred onto nitrocellulose membranes. The membranes were blocked with 5% milk in PBST and then incubated with a rabbit anti-Glut1 (Cat^#^: D160433, BBI Solutions) or rabbit anti-Glut 3 (Cat^#^: D260435, BBI Solutions), rabbit anti-human Casp 3 (Cat^#^: D220074, BBI Solutions.) or c-Myc rabbit polyclonal antibody (Cat^#^: YT0991, Immunoway) or HSP 90 (C45G5) rabbit mAb antibody (Cat^#^: 4877, Cell Signaling Technology) at 4 °C overnight. After washing with PBST, the blots were treated with a horseradish peroxidase (HRP) conjugated anti-rabbit IgG or anti-mouse antibody for 1 hr at room temperature. Detection of proteins was performed using enhanced chemiluminescence and autoradiography.

**Statistical Analysis.** The data were presented as the means ± SD from three independent experiments and evaluated through t-tests. Values of *p* < 0.05 were considered statistically significant. The analyses were performed by GraphPad Prism 5 software (GraphPad Software, San Diego, CA, USA).

## Results

### Ascorbate-induced cytotoxicity and cell cycle arrest in hepatic cells

**Figure 1:**
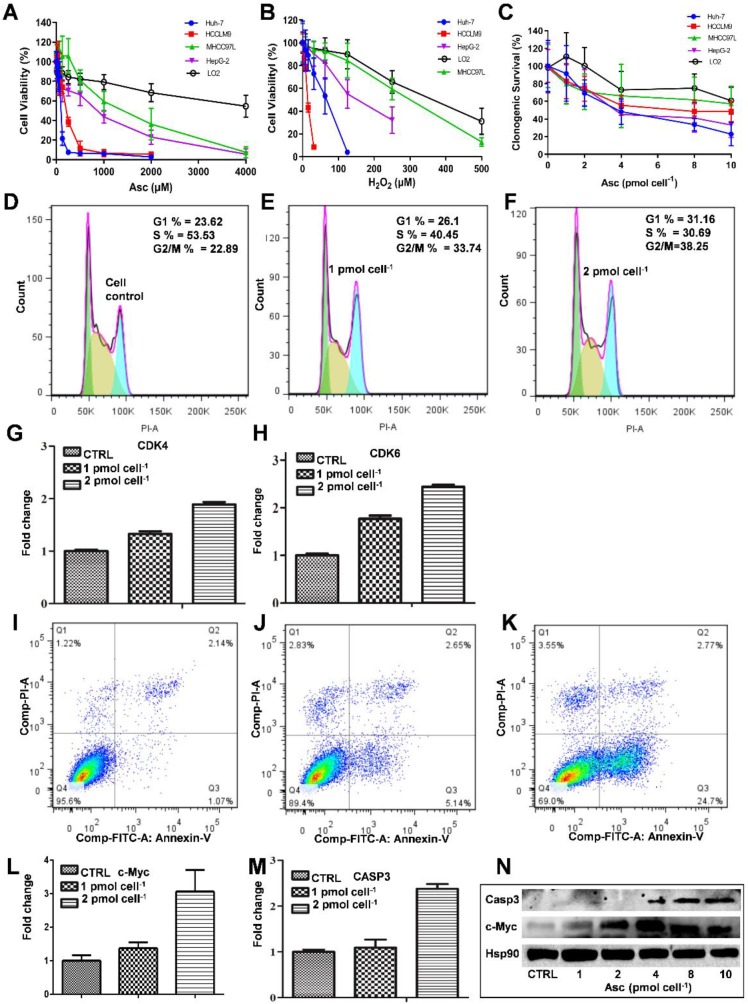
** The effect of ascorbate and H_2_O_2_ on hepatic cells.** Relative cytotoxicity of ascorbate (A, C) and H_2_O_2_ (B) on liver normal and cancer cells. Ascorbate induced G1 arrest (D-F) and apoptosis (I-K) in Huh-7 cells. qRT-PCR analysed* CDK4* (G) and *CDK6* (H), *c-Myc* (L), and *Casp3* (M) gene expression in ascorbate-treated Huh-7 cells. Immunoblots analysis of c-Myc and Casp3 expression in ascorbate-treated Huh-7 cells (N).

The MTT assay was used to detect the ascorbate-induced cytotoxicity in cancerous and non-cancerous hepatic cell lines. As demonstrated in Figure 1A-D, different liver cancer cells showed differential sensitivity to ascorbate (Figure 1A) and H_2_O_2_ (Figure 1B). The LD_50_ values of ascorbate on Huh-7, HCCLM9, HepG2 and MHCC97L cells were found to be 200 μM, 300 μM, 950 μM and 2000 μM, respectively, while the normal hepatocyte LO2 cells maintained 80% viability even at 4.0 mM ascorbate. At the same time, the LD_50_ values of H_2_O_2_ on HCCLM9, Huh-7, HepG2, MHCC97L and LO2 cells were found to be 10 μM, 55 μM, 200 μM, 320 μM and 450 μM, respectively. In the soft agar assay, P-AscH^-^ moles per cell were used to specify dose 32. Consistent with our MTT assay after one hour ascorbate treatment a differential sensitivity to ascorbate was detected in the five hepatic cell lines, Huh-7, HCCLM9, HepG2, MHCC97L, and LO2 (Figure 1C and Figure S1).

Because Huh-7 cells had the lowest LD_50_ for ascorbate, we characterized these cells more extensively. Cell cycle changes induced by ascorbate were measured by PI staining (Figure 1D-F). Ascorbate at concentrations of 2.0 pmol cell^-1^ induced a significant increase (from 23.62% to 31.16%) in the percentage of cells in the *G0/G1* phase and an even larger decrease (from 53.53% to 30.69%) in the S phase (Figure 1F) compared to no treatment (Figure 1D). qRT-PCR analysis showed that the expression of the cell cycle-related genes *CDK4* and *CDK6* in Huh-7 cells were increased by the 2.0 pmol cell^-1^ Asc treatment (Figure 1G-H). Annexin V-FITC and PI staining was applied to detect apoptosis in Huh-7 cells. As shown in Figure 1I-K, after treatment with ascorbate, apoptosis of Huh-7 cells occurred in a concentration-dependent manner. Compared with the control group, the group treated with 2.0 pmol cell^-1^ ascorbate exhibited a significantly higher percentage of apoptotic cells (Figure 1K). 24 hrs after Asc treatment apoptosis induction occurred in 24% of treated cells compared to 1% in control cells. In addition, apoptosis-related genes* c-Myc* and *Casp3* (*caspase-3*) were analysed by qRT-PCR. As shown in Figure 1L-M, the expression of *c-Myc* and* Casp3* increased in a concentration-dependent manner. Immunoblot analysis also demonstrated that Casp3 increased its expression when Huh-7 were treated with increased concentrations of ascorbate. c-Myc protein expression also increased after treatment with 4.0 pmol cell^-1^ ascorbate but decreased with 10 pmol cell^-1^ ascorbate (Figure 1N). These findings are consistent with previous studies which showed oncogenic signals such as Ras and c-myc are involved in the process of hepatocarcinogenesis and regulate the expression of metabolic enzymes which induce cancer cell death by apoptosis 35-37.

**Figure 2:**
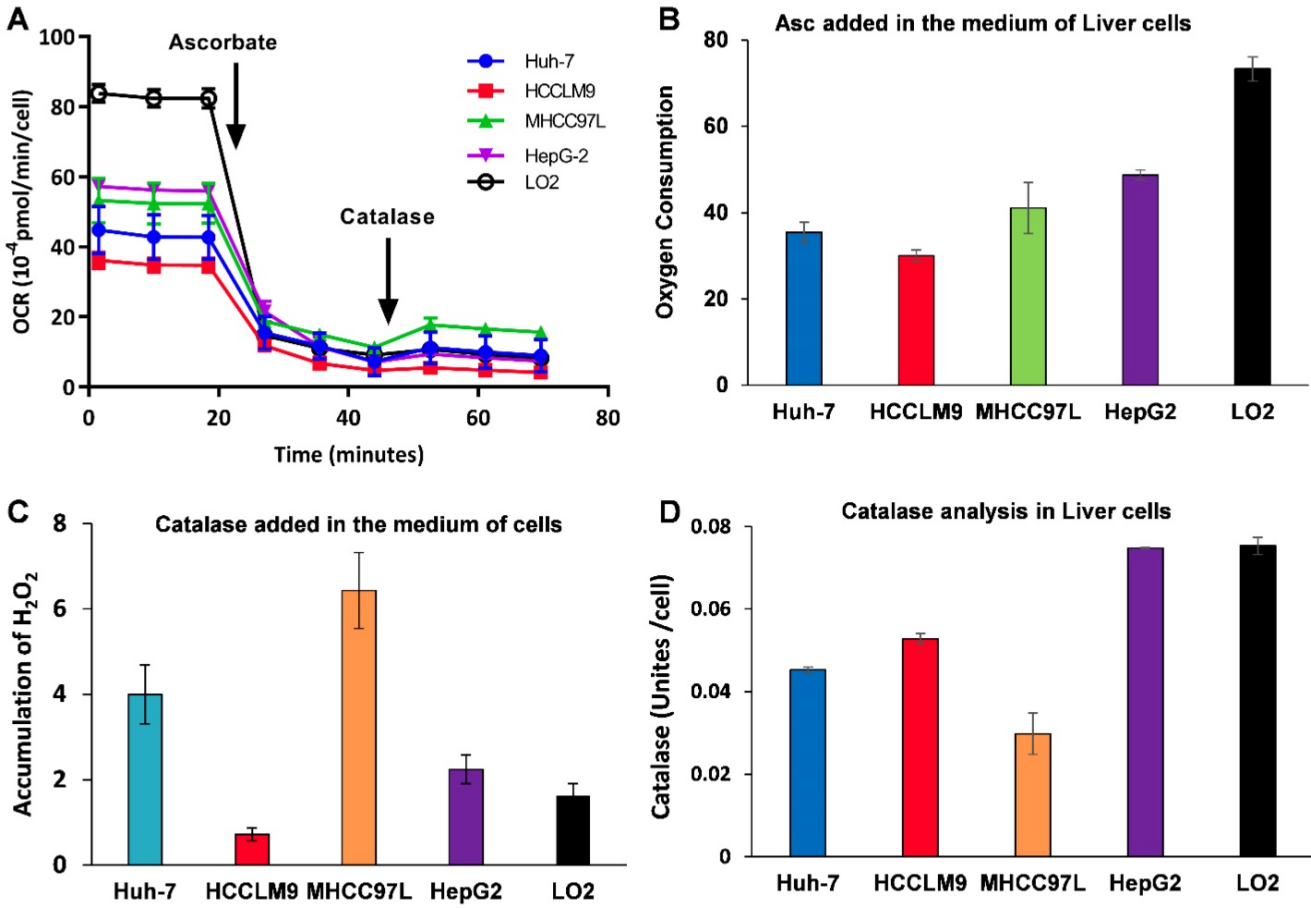
Ascorbate was oxidized in medium generating a flux of H_2_O_2_ (A-C) and catalase activity varies across cancer cell lines (D). A, B) The rate of oxygen consumption upon the introduction ascorbate (3.0 mM) in medium; C) Addition of catalase leads to a return of oxygen, which indicates that H_2_O_2_ accumulated in the medium; D) Catalase activity assay.

Because Asc oxidation can generate H_2_O_2,_ which is cyctotoxic to cancer cell 32, we hypothesized that the sensitivity of hepatic cancer cells to ascorbate was due to their lower capacity to remove extracellular H_2_O_2_. An XFe-24 Extracellular Flux Analyzer was used to measure the amount of H_2_O_2_ in complete culture medium with five hepatic cells after dissolution of ascorbate (Figure 2A-C). The rate of oxygen consumption (OCR) upon addition of ascorbate to DMEM cell culture medium provides information on the productionof H_2_O_2_ 32, 38. In our experimental setting in which 10,000 cells were treated with 3.0 mM Asc in 100 μL of DMEM medium. Addition of ascorbate to culture medium resulted in an increase in the background rate of oxygen consumption rate (Last rate measurement before ascorbate injection) - (Minimum rate measurement after ascorbate). The metabolic rates of oxygen consumption by Huh-7 and HCCLM9, MHCC97L, HepG2 and LO2 cells were 35.03, 30.03, 41.09, 48.74 and 73.28 pmol min^-1^ cell^-1^, respectively (Figure 2B), which represents the rate of H_2_O_2_ production from the oxidation of ascorbate. Next, addition of catalase indicated an accumulation of H_2_O_2_ (Maximum rate measurement after catalase) - (last rate measurement after ascorbate) in the medium over the course of an experiment. The assay revealed that the amount of H_2_O_2_ in the presence of cells, especially LO2 (1.62 pmol min^-1^ cell^-1^) and HCCLM9 cells (0.72 pmol min^-1^ cell^-1^), was less than that in HepG2 (2.25 pmol min^-1^ cell^-1^), Huh-7 (4.00 pmol min^-1^ cell^-1^) and MHCC97L (6.43 pmol min^-1^ cell^-1^) (Figure 2C). These findings indicate that ascorbate oxidation produces extracellular H_2_O_2_, the sensitivity of these hepatic cells to P-AscH^-^ is possibly due to their lower capacity to remove extracellular H_2_O_2_. Indeed, the differential sensitivity to Asc across different cancer cells was also reflected by catalase activity. We measured intracellular catalase activity in Huh-7, HepG2, HCCLM9, MHCC97L and LO2 cell lysates. We verified the catalase activity of LO2 and HepG2 cells (0.075 Unites cell^-1^) were higher than that of Huh-7 (0.045 Unites cell^-1^) and HCCLM9 (0.053 Unites cell^-1^) (Figure 2D), which was consistent with our results showing ascorbate oxidation and H_2_O_2_- induced cytotoxicity (Figure 1A-C). Our results demonstrated that extracellular ascorbate oxidation and intracellular catalase levels could be corelated to the sensitivity to P-AscH^-^ across different hepatic cells. However, we also noted that catalase activity in MHCC97L cells was less than that in other hepatic cells, which suggested other metabolic pathways might also be involved in hepatic cell sensitivity to P-AscH^-^.

### Ascorbate modifies the mitochondrial energetics of HCC cancer cells

**Figure 3:**
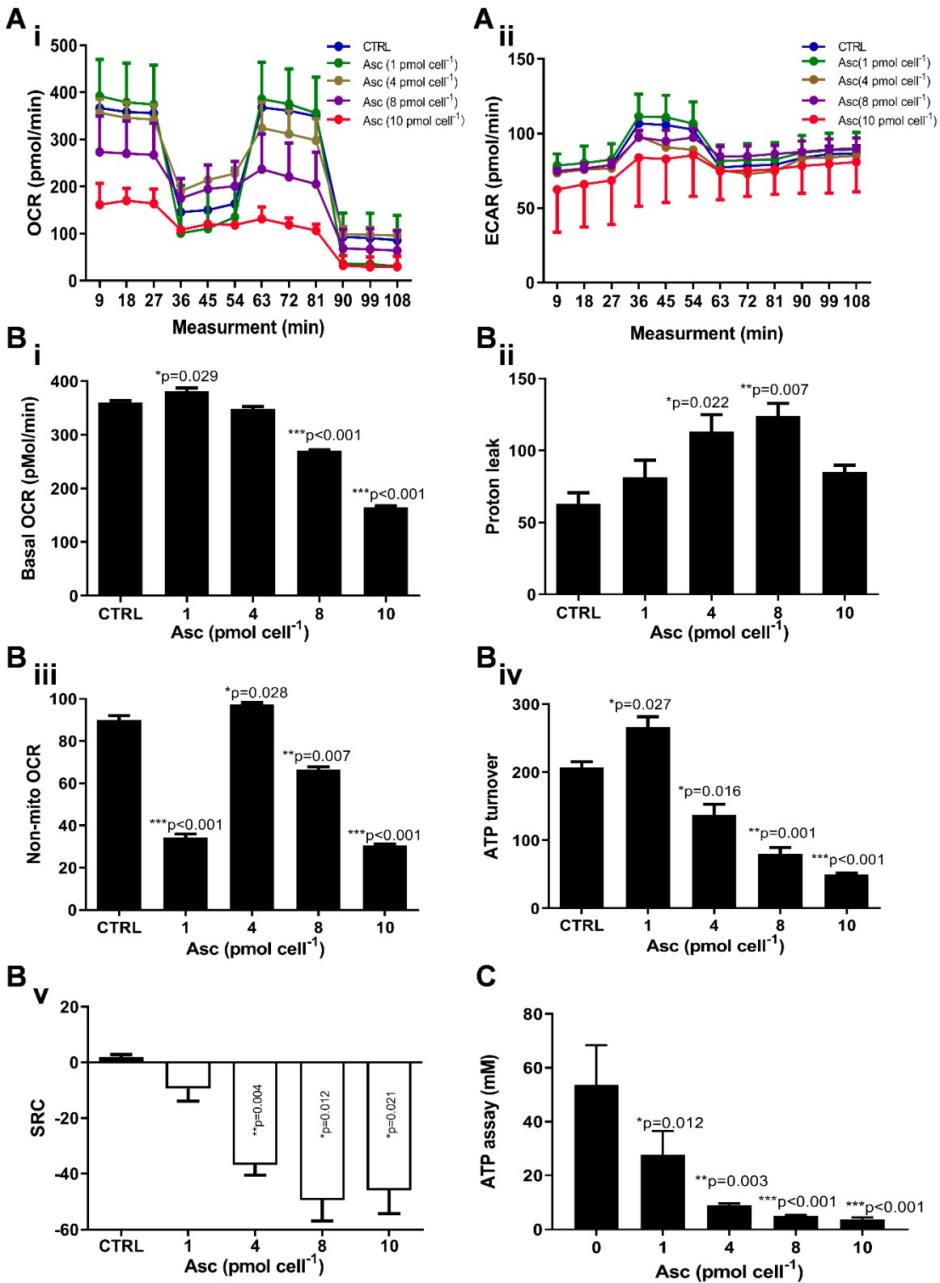
** Mitochondrial (Mito) respiration and ATP assay in Huh-7 cells treated with different doses of ascorbate (0 - 10 pmol cell-1). Ai)** Mito stress test: OCR (oxygen consumption rate); **Aii)** ECAR (extracellular acidification rate); **B)** Mitochondrial respiration parameters analysis in Mito stress test including basal OCR (i), Proton leak (ii), Non-mitochondrial respiration (iii), ATP production (iv) and SRC (spare respiratory capacity) (v); **C)** Intracellular ATP levels in Huh-7 cell by ATP Assay Kit; One-way ANOVA p values are shown to determine the significance across different doses. The significance between CTRL and other time points was determined by subsequent unpaired t-tests. *p<0.05, **p<0.01, and ***p<0.005

**Figure 4:**
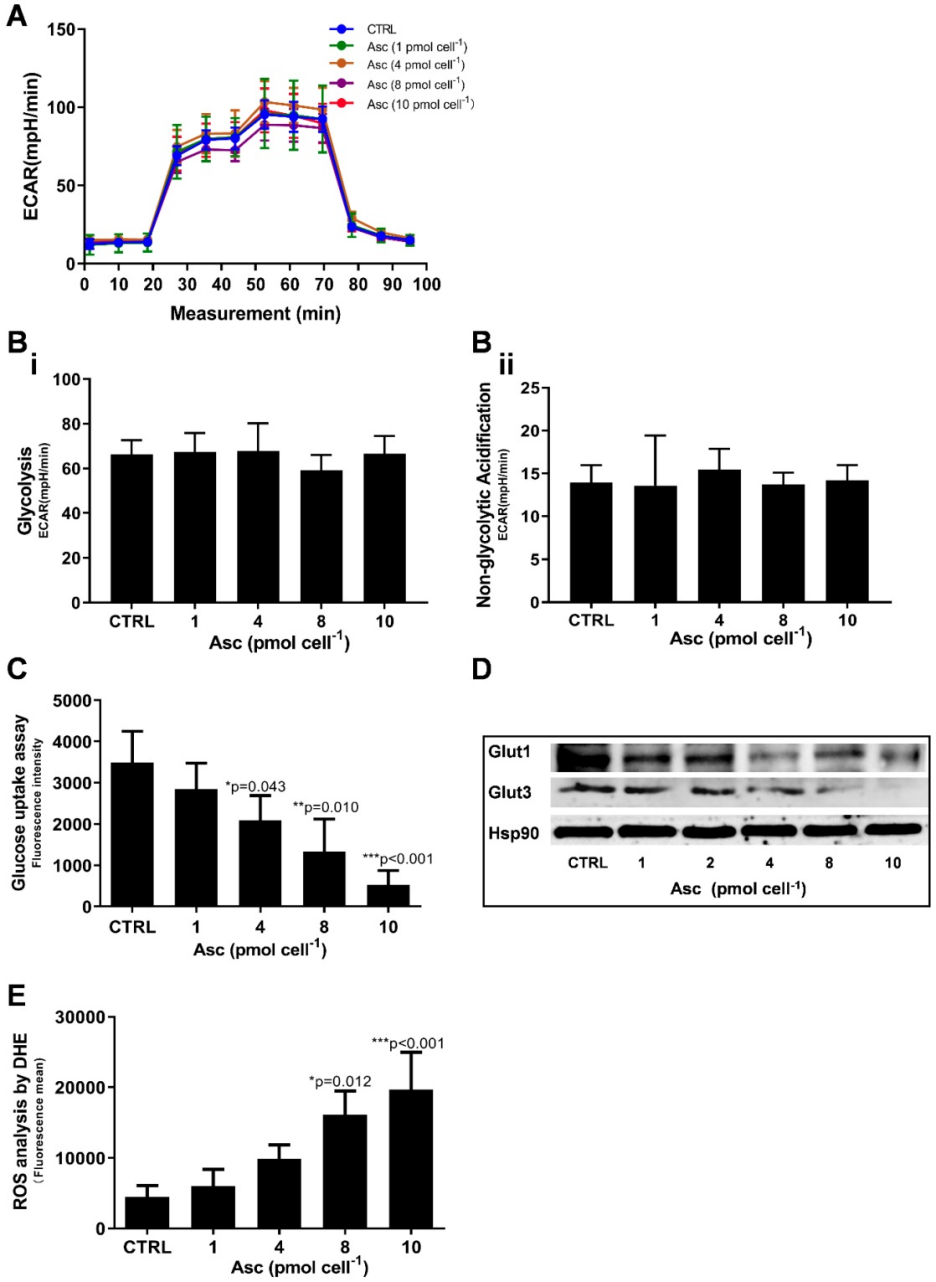
** Glycolysis assay, ROS production and glucose uptake analysis in Huh-7 cells treated with different doses of ascorbate (0 - 10 pmol cell^-1^). A)** ECAR curve by Glycolysis stress test; **B)** Glycolysis parameters analysis in Glycolysis stress test including glycolysis capacity (i) and Non-glycolytic Acidification (ii); **C)** Fluorescence intensity changes of 2-NBDG in cells for glucose uptake analysis; **D)** Immunoblots analysis of Glut1 and Glut3 expression; E) ROS analysed in Huh-7 treated with P-AscH by DHE; One-way ANOVA p values are shown to determine the significance across different doses. The significance between CTRL and other time points was determined by subsequent unpaired t-tests. *p<0.05, **p<0.01, and ***p<0.005

We then hypothesized that ascorbate cytotoxicity on Huh-7 cells is mediated by altering mitochondrial respiration. To test this possibility, we compared the changes in oxygen consumption rate, spare respiratory capacity (SRC), and extracellular acidification rate (ECAR) in Huh-7 cells in response to different ascorbate doses. Mitochondrial parameters in Huh-7 cells were monitored using a Seahorse XFe-24 oxygen and proton flux analyser after treatment with 0 - 10 pmol cell^-1^ ascorbate for 1 hr. A biphasic phenotype of mitochondrial energetics was induced by the different ascorbate concentrations (Figure 3A). Comparing to the control cells, low-dose (1.0 pmol cell^-1^) ascorbate significantly increased in OCR at 25.41 pmol min^-1^ (*p* = 0.029) (Figure 3Bi) and ATP production (*p* = 0.027) (Figure 3Biv) but decreased in extramitochondrial OCR (*p* < 0.001) (Figure 3Biii), whereas mediate-dose (4.0 pmol cell^-1^) ascorbate depressed mitochondrial respiration with a significant reduction in ATP production (*p* = 0.016) (Figure 3Biv) and SRC (*p* = 0.004) (Figure 3Bv) but increased proton leakage (*p* = 0.022) (Figure 3Bii) and extramitochondrial OCR (*p* = 0.028) (Figure 3Biii); high doses (8.0 pmol cell^-1^ or 10 pmol cell^-1^) of ascorbate reduced mitochondrial respiration by minimizing OCR (*p* < 0.001) (Figure 3Biii), SRC (*p* = 0.021) (Figure 3Bv) and ATP production (*p* < 0.001) (Figure 3Biv). ATP analysis of Huh-7 cells treated with ascorbate (0 - 10 pmol cell^-1^) also verified that P-AscH^-^ decreased the intracellular concentration of ATP in Huh-7 in a dose-dependent manner (Figure 3C). Surprisingly, in Huh-7 cells ascorbate appeared to have no effect on glycolysis; neither low- nor high-dose ascorbate significantly altered the extracellular acidification rate (ECAR) in Mito stress test (Figure 3Aii) and Glycolysis stress test (Figure 4A). Noticeably, ascorbate could selectively kill KRAS mutant cancer cells by downregulating Glut1 expression to affect glucose consumption, but had no significant effect on the glycolytic rate in KRAS cells or BRAF Wild type (WT) cells 35, 36, similar to our results of ECAR in Huh-7 cells treated with different P-AscH^-^ which have demonstrated that the glycolysis (Figure 4 Bi) and extracellular acidification rate (Figure 4 Bii) and were not significantly difference in ECAR (all* p* > 0.05). Previous studies have shown there was a remarkably low K-Ras rate or no BRAF mutation in hepatocellular carcinoma cells 39, 40 and the mutations of KRAS and BRAF were not present in Huh-7 cells (http://amp.pharm.mssm.edu/Harmonizome/gene_set/HUH7/CCLE+Cell+Line+Gene+Mutation+Profiles), which might explain why glycolysis, as measured by our ECAR assay, was unaffected by P-AscH^-^ in Huh-7 cells. As illustrated in Figure 4C, glucose uptake was reduced in Huh-7 cells treated with P-AscH^-^ and the fluorescence intensity decreased gradually when the ascorbate concentration was gradually increased from 4.0 pmol cell^-1^ to 10 pmol cell^-1^ with respect to the cell controls without ascorbate. To determine whether the changes in glucose transport occurred at the level of protein expression, we analysed the expressions of Glut1 and Glut3. Immunblots revealed that the expression levels of Glut1 and Glut3 were decreased in Huh-7 cells treated with P-AscH^-^, when compared to controls without ascorbate (Figure 4D).

As integrated mitochondrial respiration is closely associated with the production of reactive oxygen species (ROS), modifications of OCR and/or mitochondrial coupling (proton leak) may change the dynamics of ROS generation. We thus examined the effect of ascorbate on intracellular superoxide production using the oxidation-sensitive fluorescent probe DHE. As shown in Figure 4E, after treatment with ascorbate, the intracellular superoxide production was dramatically increased in Huh-7 cells treated with 8.0 pmol ascorbate per cell (*p* = 0.012) in a concentration-dependent manner. Collectively, these data indicated that ascorbate alters the mitochondrial bioenergetics of Huh-7 cells in a dose-dependent manner. Low-dose ascorbate boosts and high-dose depresses oxidative phosphorylation, which implied that the dose-dependent effects of ascorbate on the activation/depression of oxidative pathway might be associated with ROS generation.

### Pharmacologic ascorbate inhibited HCC growth in a mouse model

**Figure 5:**
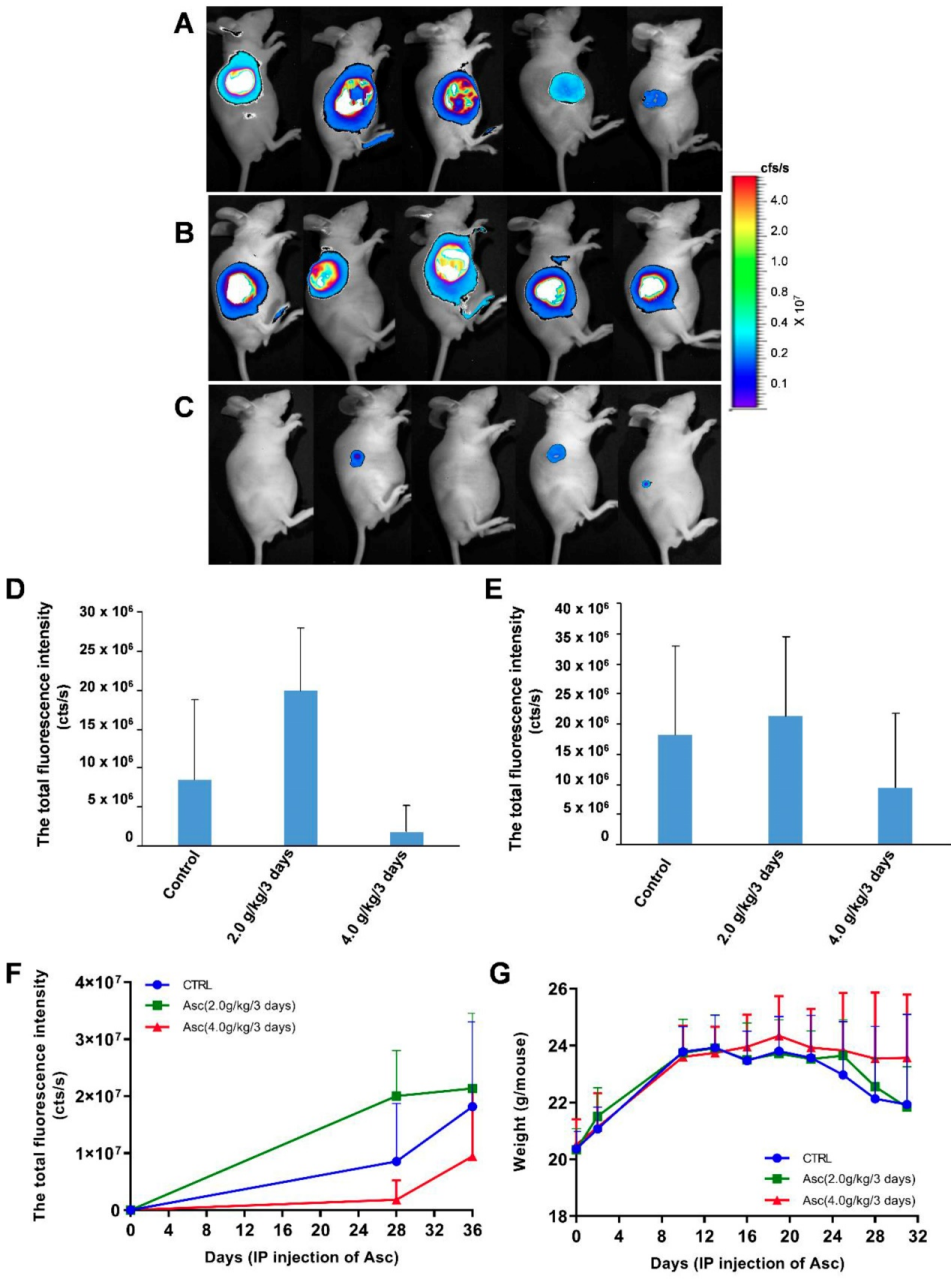
** The effect of xenograft tumour mice treated with ascorbate. A-C)** The growth and volume of xenograft tumours in mice treated with ascorbate were measured after 6 weeks by bioluminescence imaging: A) Control (PBS); B) Ascorbate at 2.0 g/kg/3 days; C) Ascorbate at 4.0 g/kg/3 days. **D-F)** Bioluminescence imaging analysis of the total fluorescence intensity in mice with xenograft tumours treated with ascorbate on Day 28 (D) and Day 35 (E) after IP injection of ascorbate; G) The weights of mice with xenograft tumours treated with ascorbate.

For the xenograft tumour mouse model *in vivo* studies, we chose Huh-7 cells because of their sensitivity to ascorbate. To determine if pharmacologic ascorbate inhibits HCC growth, we treated the xenograft tumour model of Huh-7 cells bearing lentivirus-luciferase with either PBS, 2.0 g/kg/3 days and 4.0 g/kg/3 days of ascorbate, respectively. Tumour volumes were then measured after 5 weeks of treatment by bioluminescence imaging. We found tumour growth was significantly reduced with IP injection of ascorbate at 4.0 g/kg/3 days on Day 28 (Figure 5D) and Day 35 (Figure 5E) (compared to the tumour growth in the PBS control group (Figure 5F). Huh-7 tumour volume in mice treated with a higher dose of ascorbate (4.0 g/kg/3 days IP) was decreased by 48.09% compared to the tumour volume in control mice (PBS) (*p* < 0.05). However, Huh-7 tumour volume in mice treated with IP injection of ascorbate at 2.0 g/kg/3 days was increased by 17.53% compared to the volume of control tumours (PBS) (*p* < 0.05) (Figure 5E-F). HCC mice given IP injection of ascorbate at 4.0 g/kg/3 days weighed significantly more than HCC mice given IP injection of ascorbate at 2.0 g/kg/3 days and their controls (PBS) (23.5 g *vs.* 22.6 g, respectively, *p* < 0.05; 23.5 g *vs.* 22.1 g, respectively, *p* < 0.05) after 6 weeks. However, HCC mice given IP injection of ascorbate at 2.0 g/kg/3 days weighed no significantly differently to their controls (PBS) (22.6 g *vs.* 22.1 g, respectively, *p* = 0.69) (Figure 5G).

### Gene expression profiling and pathway analysis of HCC tumour tissue from mice given IP injection of ascorbate at 4.0 g/kg/3 days

To further corroborate the possible molecular mechanism of HCC treated with ascorbate, we analysed the genome-wide mRNA expression profiles in nine HCC tumour tissue samples from three mice treated with IP injection of ascorbate at 4.0 g/kg/3 days, three mice treated with IP injection of ascorbate at 2.0 g/kg/3 days, and three control mice (only PBS, without ascorbate IP). Using a normal cut-off value greater than two-fold and a *P* value at or below 0.05, we identified changes in transcript levels of 1632 and 1501 genes/ncRNAs in HCC tumour tissue from mice treated with IP injection of ascorbate at 4.0 g/kg/3 days and 2.0 g/kg/3 days compared with expression in their controls, respectively (Data not shown). Subsequently, a Kyoto Encyclopaedia of Genes and Genomes (KEGG) analysis was performed to determine the top 30 pathways of the differential mRNAs. Nineteen common pathways were identified in mouse HCC tumour tissue treated with IP injection of ascorbate at 4.0 g/kg/3 days and 2.0 g/kg/3 days, including Type II diabetes mellitus, Type I diabetes mellitus, TGF-beta signalling pathway, Staphylococcus aureus infection, Rheumatoid arthritis, Protein digestion and absorption, Prion diseases, NOD-like receptor signalling pathway, Malaria, Leishmaniasis, Intestinal immune network for IgA production, Fatty acid degradation, ECM-receptor interaction, Dilated cardiomyopathy, Complement and coagulation cascades, Antigen processing and presentation, Amoebiasis, Allograft rejection, and African trypanosomiasis (Figure S2A-B). In HCC tumour tissue from mice treated with IP injection of ascorbate at 2.0 g/kg/3 days compared with expression in their controls. Comparative analysis of the genome-wide mRNA expression profiles from HCC mouse HCC tumour tissue treated with IP injection of ascorbate at 4.0 g/kg/3 days and 2.0 g/kg/3 days identified several metabolism and cell differentiation pathways, such as Vitamin digestion and absorption, Toll-like receptor signalling pathway, Renin-angiotensin system, Phagosome, Osteoclast differentiation, Maturity onset diabetes of the young, Linoleic acid metabolism, Glycosphingolipid biosynthesis, Glycosaminoglycan biosynthesis, Fat digestion and absorption, alpha-Linolenic acid metabolism were only included in the top 30 of GO analysis of mouse HCC tumour tissue treated with IP injection of ascorbate at 2.0 g/kg/3 days (Figure S2A). Importantly, 192 genes/ncRNAs were uniquely differentially expressed in HCC tumour tissue obtained from mice treated with IP injection of ascorbate at 4.0 g/kg/3 days (not at 2.0 g/kg/3 days) when compared with expression in controls (Table S2). The microarray experiments revealed that the following genetic networks are altered in HCC treated with high-dose ascorbate: Hereditary Disorder, Nutritional Disease, Organismal Injury and Abnormalities; Cancer, Cellular Movement, Organismal Injury and Abnormalities; Haematological System Development and Function, Immune Cell Trafficking, Inflammatory Response; Connective Tissue Disorders, Organismal Injury and Abnormalities, Skeletal and Muscular Disorders; and Cell-To-Cell Signalling and Interaction, Behaviour, Digestive System Development and Function (Table 1). The genes deregulated in HCC treated with high-dose ascorbate belonged to five canonical signalling pathways that are frequently deregulated in cancer: Insulin Receptor Signalling, Dolichol and Dolichyl Phosphate Biosynthesis, UDP-N-acetyl-D-glucosamine Biosynthesis II, Ceramide Biosynthesis, and IL-3 Signalling Pathways (Table S3). Although seven genes (*SCNN1A, PPP1R14C, BAD, INPP5D, DHDDS, FMO3,* and* SPTSSB*) were represented in the signalling pathways, each of these seven genes is functionally involved in insulin receptor signalling and metabolism and has been implicated to mitochondrial respiration in the treatment of HCC with high-dose ascorbate 41, 42.

### AGER, DGKK, ASB2, TCP10L2, Lnc-ALCAM-3, and Lnc-TGFBR2-1 expression were altered in HCC tumour tissue from mice treated with high-dose ascorbate

**Figure 6:**
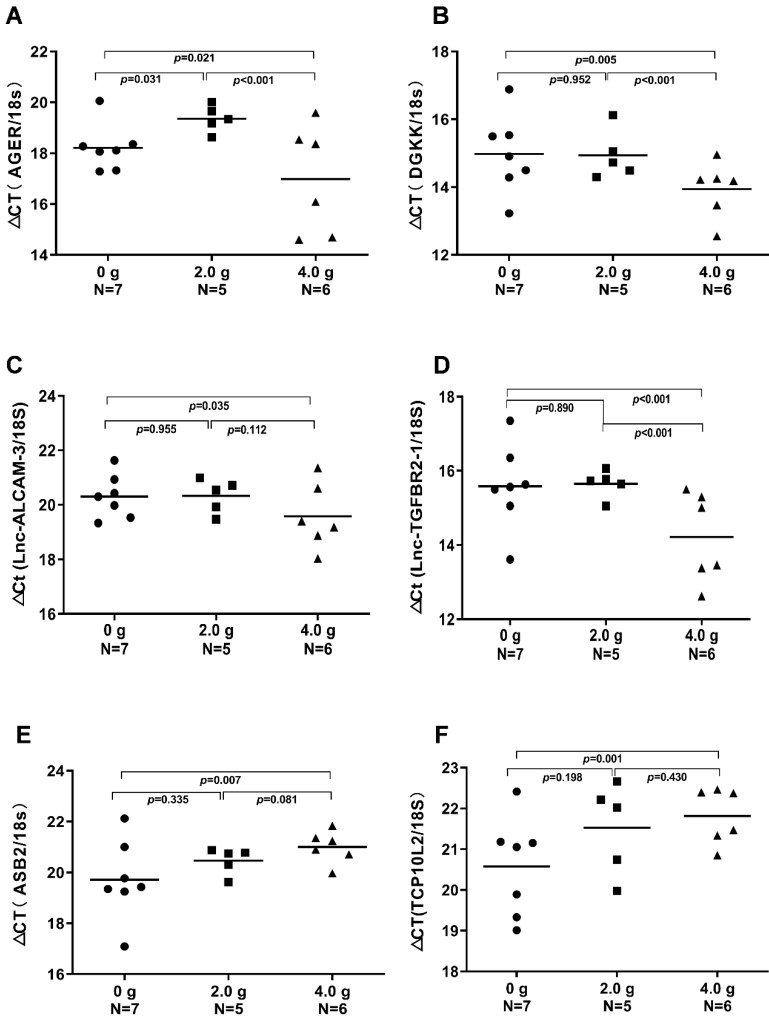
**Real-time PCR (qRT-PCR) analyses of *AGER, DGKK, ASB2, TCP10L2.******Lnc-ALCAM-3*****, and *Lnc-TGFBR2-1*** mRNA expression in xenograft tumour mice treated with ascorbate, which were evaluated through t-tests. **A)** ΔCt (*AGER /18S*); **B)** ΔCт (*DGKK /18S*); **C)** ΔCt (*Lnc-ALCAM-3 /18S*); **D)** ΔCt (*Lnc-TGFBR2-1 /18S*); **E)** ΔCт (*ASB2 /18S*); **F)** ΔCt (*TCP10 L2 /18S*).

**Figure 7:**
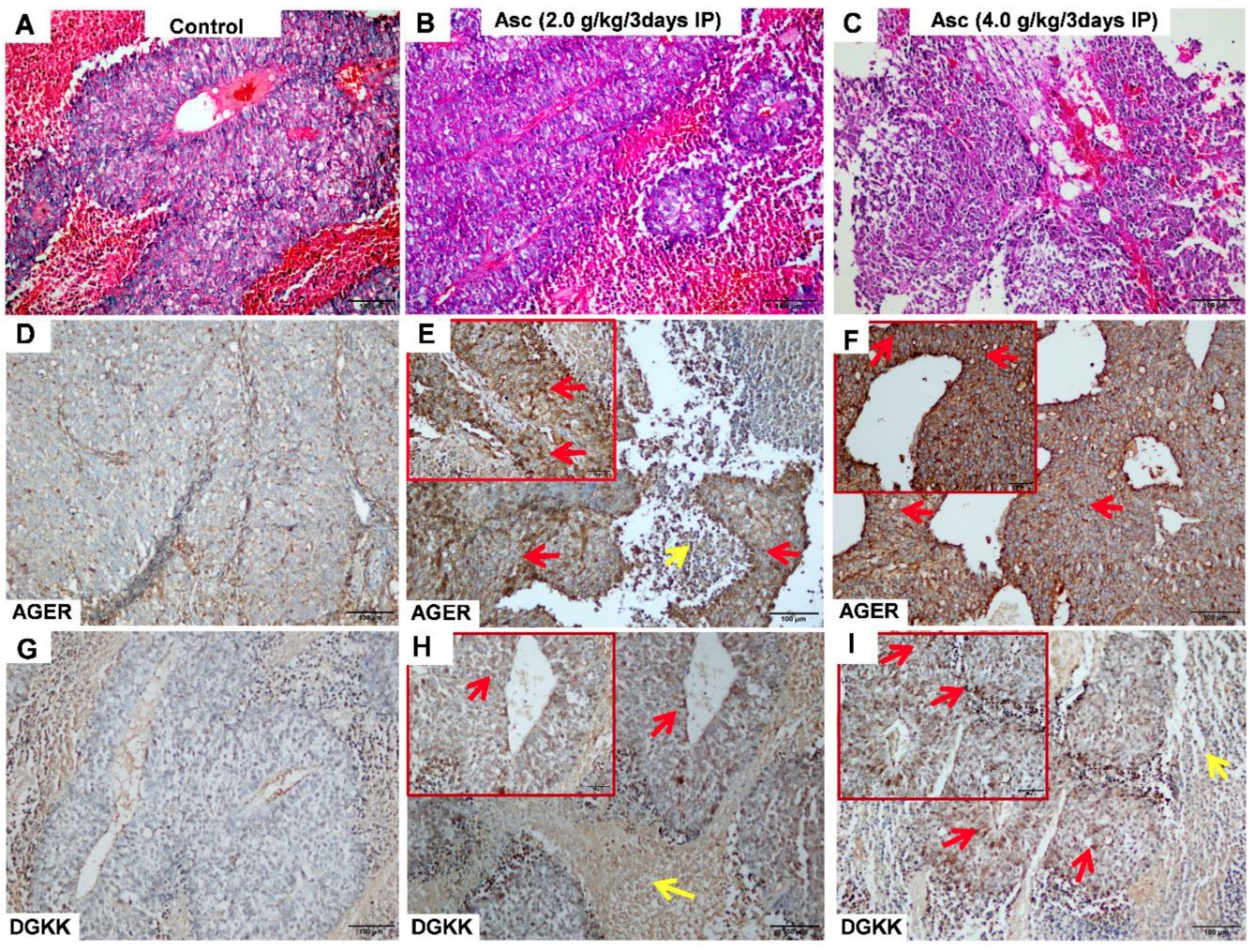
**Immunohistochemical (IHC) analysis of AGER (D-F) and DGKK (G-I) expression with HE staining (A-C) in xenograft tumour mice treated with ascorbate. A, D, G)** Control (PBS); **B, E, H)** ascorbate (2.0 g/kg/3 days); **C, F, I)** ascorbate (4.0 g/kg/3 days). The staining of AGER and DGKK in hepatoma cells is shown with red arrows, whereas yellow arrows represent necrosis cells.

We validated the gene expression levels of *AGER, DGKK, ASB2, TCP10L2, Lnc-ALCAM-3*, and* Lnc-TGFBR2-1* in HCC tumour tissue samples from mice treated with high-dose ascorbate by qPCR. Consistent with array data, qRT-PCR analyses of *AGER/RAGE* (Advanced Glycosylation End-Product Specific Receptor; Receptor For Advanced Glycosylation End Products) and *DGKK* mRNA expression levels revealed a 5.08-fold (*p* = 0.020) and 2.24-fold (*p* = 0.005) increase in *AGER/RAGE* and *DGKK* gene expression, respectively, in HCC tumour tissue samples from mice treated with high-dose ascorbate showed when compared to controls, and a 11.35-fold (*p* < 0.001) and 2.47-fold (*p* = 0.001) increase in* AGER/RAGE* and *DGKK* gene expression, respectively, in HCC tumour tissue samples treated with low-dose ascorbate compared to controls respectively, when compared to controls (Figure 6A-B). *Lnc-ALCAM-3* and *Lnc-TGFBR2-1*) gene expression levels were increased in HCC tumour mice treated with high-dose Asc by (2.19 fold) (*p* = 0.035) (2.73 fold) (*p* < 0.001) respectively, when compared to controls, and by 2.05-fold (*p* = 0.111) and 3.61-fold (*p* < 0.001) respectively in HCC tumour mice treated with low-dose ascorbate compared with controls were (Figure 6C-D). The repression of *ASB2* and *TCP10L2* gene expression levels in HCC tumour mice treated with high-dose ascorbate compared to control was also verified. (Figure 6E-F). To further investigate the expression of AGER/RAGE and DGKK proteins in HCC tumour mice treated with high (4.0 g/kg/3d) and low-dose ascorbate (2.0 g/kg/3d), we performed immunohistochemical (IHC) staining of 18 tumour tissue samples (Figure 7) and observed weak membranous staining of AGER/RAGE in hepatoma cells and low-dose ascorbate tissue samples (Red arrow) (2.0 g/kg/3d IP), with the yellow arrow representing the area of ​​necrosis (Figure 7C-E). The diffuse and strong membranous staining of AGER/RAGE in high-dose ascorbate samples (4.0 g/kg/3 days IP) showed higher expression of the protein (Figure 7F). Weak cytoplasmic and nuclear staining of DGKK in hepatoma cells and in the low-dose ascorbate samples (2.0 g/kg/3d IP) is shown by the red arrows, whereas the yellow arrows represent the area of ​​necrosis (Figure 7H). DGKK was strongly expressed in the cytoplasm of cancer cells and co-expressed in the nucleus and cytoplasm of few hepatoma cells in high-dose ascorbate samples (4.0 g/kg/3 days IP) (Figure 7I). Taken together, stronger staining with both anti- AGER/RAGE and -DGKK antibodies was observed in HCC cancer cells treated with high-dose ascorbate (4.0 g/kg/3 days IP) (Figure 7).

## Discussion

Pharmacological ascorbate may represent an easily implementable drug as non-toxic adjuvant to conventional HCC treatments. To date there have been two case studies involving HCC patients and high dose IV ascorbate. One was a patient with a hepatocellular carcinoma with a remarkable response to high dose IV ascorbate that we received from clinicians in Malaysia (Dr. Raymond Ngeh and Dr. Robert Luk Clinical notes). The patient was a middle-aged woman with massive primary HCC. The cancer specialist suggested no further treatment as she will die in a few weeks. On her pre-treatment CT scan, the patient was given high dose IV vitamin C (1.5 - 2.0 gram per kg body weight) and low dose chemotherapy which included 3 generic chemotherapy drugs at 1/3 usual dose (HiCLoChemo), PET/CT scan images of the patients after 4 cycles of HiCLoChemo treatment, compared with pre-treatment PET/CT scan were presented in the Figure S3. The HCC cancer is now in remission, and the patient remains otherwise healthy. In the second case report the patient presented with a metastatic disease to the left 8th rib and his medical oncologist determined that the patient was a candidate for standard of care chemotherapy with sorafenib along with 75 grams IV ascorbic acid administered three times per week. Before high dose IV vitamin C treatment, he had a left 8th rib metastasis (74 × 44 mm), along with a 2.8 × 2.2 cm ablation cavity from premetastatic treatment in the right lobe of the liver After 16-week cycles of ascorbate and sorafenib, his rib lesion was markedly smaller (43 × 28 mm), with no change in the liver function and no suggestion of additional metastasis. They also demonstrated that ascorbate could act synergistically with sorafenib in killing HepG2 cells involving the dysregulation of cellular calcium homeostasis 20. The above two cases indicate that high dose IV ascorbate shows strong promise for the treatment of HCC in humans, and highlighted a plausible mechanism of the anti-tumour activity.

Although most animals and plants can synthesize ascorbate through a sequence of enzyme-driven steps involving conversion of monosaccharides to ascorbate (Figure S4), some animals and human cannot synthesize ascorbate because of the lack of the functional L-gulonolactone oxidase (GULO) 43. Importantly, ascorbate produces extracellular hydrogen peroxide involving redox-active labile iron and induces DNA damage and ATP depletion in CC, colon, NSCLC and GBM cancer cells 8, 10, 11, 44, which implies that cancer cells treated with high-dose ascorbate can utilize a metabolic pathway in the hypoxic environment of hepatoma. We therefore compared the changes in OCR, ECAR and SRC in Huh-7 cells in response to ascorbate treatment. Interestingly, low-dose (1.0 pmol cell^-1^) ascorbate promoted Huh-7 mitochondrial respiration with significant increases in OCR and ATP production but decreases in extramitochondrial OCR when compared to control untreated cells. These results are in accordance with previous studies where patient fibroblasts treated with ascorbate exhibited significantly higher ratios of complexes I-III and II-III in comparison to the same cell lines without ascorbate treatment 45. High-dose (4.0 pmol cell^-1^) ascorbate however produced biphasic phenotype in mitochondrial energetics: mitochondrial respiration, ATP production and SRC were significantly reduced, but proton leakage, extramitochondrial OCR and intracellular ROS were increased. Due to essentiality of extracellular H_2_O_2_ and independent of mitochondria dysfunction, peroxide may trigger gene expression that leads to cell death; these data indicate high doses of ascorbate served as a pro-drug to kill hepatoma by altering mitochondrial respiration.

Our main findings of the present study are that a high dose of ascorbate (4.0 g/kg/3 days) given by IP injection significantly repressed tumour growth, while a lower dose of ascorbate (2.0 g/kg/3 days) did not repress tumour growth of HCC. Given the pharmacokinetics of ascorbate, our results demonstrated that the pharmacological dose of ascorbate by IP injection was of critical importance with high doses of ascorbate inhibiting tumour growth whereas low doses act as a tumour growth factor. Our results of IP injection are in accordance with previous studies where the oral supplementation of ascorbate had no anticancer effect 5-7, and the parenteral administration of high-dose ascorbate can significantly inhibit tumour growth in* TLT* (*TREM*-like transcript)-bearing mice 46. However, the early phase research of ascorbate treatment was inappropriately performed, and the various parameters in these clinic studies (doses, routes of administration, optimal schedule) were not correctly defined or standardized, leading to mixed results and controversy 47. Several papers have studied pharmacologic ascorbate in hepatoma using *in vitro* models 7, 20, 48. In mice with the hepatoma parenteral administration of ascorbate either IV or IP 1.0 g/kg of sodium ascorbate achieved pharmacologic concentrations of ascorbate in blood (>1.0 mM), whereas oral administration of the same dosage did not 7. Only in parental administration was the growth rate of a murine hepatoma decreased, resulting in pharmacologic concentrations of ascorbate that exhibit interesting anticancer properties. A key advantage of IV ascorbate over conventional chemotherapeutic agent is its lack of toxicity. Because of these properties *in vivo* IV treatment would require less frequency. One studies where ascorbate frequency of one or two treatments with intravenous administration of ascorbate per week was similar to or less than ours 16. Most animal tumours need daily or even twice daily treatment. If treatment is needed only every three days, this may mean that hepatoma in humans will be responsive to ascorbate at much less frequent dosing than is currently used for other tumours. The dosing frequency is a real clinical problem. If we have a cancer that requires less frequency, i.e. once weekly vs 3 x weekly, this means the treatment is more likely to be tested in people and actually used: the special sensitivity of human hepatoma to IV ascorbate and the need for less frequent treatment is definitely an advantage.

To determine the mechanistic basis of this phenotype, we analysed the gene expression profiles of HCC tumours from mice treated with IP injection of ascorbate at 4.0 g/kg/3 days using gene arrays and identified changes in transcript levels of 192 genes/ESTs. The upregulation in* AGER* and *DGKK* gene expression was validated by qRT-PCR and IHC analysis.* RAGE*, a member of the immunoglobulin protein family, is a multiligand receptor able to bind to advanced glycation end products (*AGEs*), which are heterogeneous, reactive, and irreversibly crosslinking molecules formed primarily by the non-enzymatic reaction of sugars with proteins 49, 50. *AGEs* can induce the apoptosis of endothelial progenitor cells (EPCs) by activating *RAGE*. Dysregulation of the *AGEs/RAGE* axis in EPCs may promote atherosclerosis and the *NADPH/ROS/JNK* signalling axis may serve as a potential target of the clinical treatment of atherosclerosis 51, 52. *AGE* and *LPS* increase *IL-6* secretion depending on *NF-κB* activation and ROS production through *RAGE* 53. *RAGE* stimulation also induces the generation of ROS mainly through the activity of NADPH oxidases. ROS accumulation has been linked to the formation of *AGEs* in various diabetic tissues 50. When cells are unable to properly adapt to ROS generation, *RAGE* activation induces oxidative stress, leading to neuronal not cancer cell death 52. *DGKK* (Diacylglycerol Kinase Kappa) was repressed in preputial tissues in carriers of the risk allele rs1934179 54, 55. Treatment with H_2_O_2_ can result in the generation of diacylglycerol (*DAG*) and *IP3*, which could increase intracellular calcium and activate several forms of *PKC*, leading to *Ras* and *Raf* activation 56-58.

Pathway analysis of our gene array data identified five canonical signalling pathways dysregulated in cancer, namely, Insulin Receptor Signalling, Dolichol and Dolichyl Phosphate Biosynthesis, UDP-N-acetyl-D-glucosamine Biosynthesis II, Ceramide Biosynthesis, and *IL-3* Signalling pathways. Ascorbate attenuated upstream hepatic insulin action and affected hepatic insulin signalling by impairing the phosphorylation and activation of the insulin receptor and its subsequent substrates, which was found in rats 59. Ascorbate suppresses the *IL-3*-induced phosphorylation of MAP kinase, which may modulate *IL-3*-mediated cytokine responses and therefore play a role in controlling inflammatory responses 60. Ceramides can induce cell apoptosis by altering the cellular redox status to be responsible for signal transduction with ROS of many extracellular agents 61. The ascorbate-mediated production of oxidative stress has been shown to retard the tumour growth of HCC, which remains the predominant mechanism behind the cellular effects of ascorbate 20, 62. Ascorbate can induce the production of ROS, leading to oxidative stress and cell death, a death pathway that is interesting if we consider the multiple apoptotic defects usually exhibited by cancer cells.

In summary, our *in vitro* studies have shown hepatic cancer cell death from pharmacologic levels of ascorbate are mediated by hydrogen peroxide, and our *in vivo* studies have shown IV ascorbate slows tumour growth via mitochondrial damage and multiple gene transcription changes. Our findings support the need for further detailed dose-response studies of ascorbate in an appropriate animal cancer model. The clinical efficacy of high-dose ascorbate in HCC treatment also needs to be explored.

## Supplementary Material

Supplementary figures and tables.

## Figures and Tables

**Table 1:** The top five networks of the 192 DEGs/ncRNAs in HCC mice treated with a high dose of ascorbate by IPA analysis.

ID	Analysis	Molecules in Network	Score	Focus Molecules
1	Hereditary Disorder, Nutritional Disease, Organismal Injury and Abnormalities	*ABCC2, ADGRF1, Akt, ANXA13, ASB2, CASP14, caspase, CHRND, CRLF1, DNMT3A, EDA, ENPP2, ERK, FMOD, FOXF2, GPR63, GPR75, GPR78, GPR176, HAMP, Histone h3, Insulin, Jnk, NFkB (complex), NKD1, NMUR1, P2RY10, Pkc(s), PPP1R14C, RNF112, S100A12, SLC52A1, Ubiquitin, USP2, Vegf*	33	16
2	Cancer, Cellular Movement, Organismal Injury and Abnormalities	*ACAN, B4GALNT1, CHGA, CLDN7, CPS1, CST2, DEFB121, EDN1, FAM81A, HSPA2, KCNJ1, KCNJ8, KRTAP10-3, MITF, MOV10 L1, NUPL2, PSMG2, PTOV1, PYGO2, RGPD4 (includes others), RNF175, SLC8A2, SMTNL2, SPATA8, STARD8, STX19, TBK1, TCP10/TCP10 L2, TMCC2, TMF1, TSFM, UBC, VEGFA, WFDC10B, YWHAZ*	31	15
3	Haematological System Development and Function, Immune Cell Trafficking, Inflammatory Response	*AGER, AMPK, AQP4, BAD, CD3, EBI3, EFNB3, ERK1/2, FMO3, Gm-csf, Hsp70, IgG, IL12 (complex), Immunoglobulin, INPP5D, Interferon alpha, KLRG1, LDL, Mapk, P38 MAPK, p85 (pik3r), PI3K (complex), Pka catalytic subunit, Pro-inflammatory Cytokine, Ras, RET, SAA, SAG, SCNN1A, SPRR2C, SRC (family), TCR, Tgf beta, Tnf (family), VCAM1*	26	13
4	Connective Tissue Disorders, Organismal Injury and Abnormalities, Skeletal and Muscular Disorders	*ACTA2, ALPL, ATXN2, C2orf40, CALCR, COL4A1, CTNNB1, DKK2, DKK3, DMKN, Ecm, F8A1 (includes others), FAM173A, FOS, FOXF2, GABRA4, GABRD, HCST, Hd-neuronal intranuclear inclusions, KREMEN, KREMEN1, MT1A, MUC6, PAFAH1B2, RNA polymerase II, S100A11, SH3GL3, SPATA22, SRF, TGFB1, TMSB15A, TP53, VTRNA1-1, VTRNA1-2, VTRNA1-3*	26	13
5	Cell-To-Cell Signalling and Interaction, Behaviour, Digestive System Development and Function	*ADCYAP1, AGER, Agtr1b, ARG1, Camk, CARTPT, CHGA, CHGB, CLCF1, CNKSR1, CREB1, CRTC1, DGKK, DLL3, DOK4, EBF3, GALR1, GFRA1, IRS, MAPK1, MAPK4, MC4R, MCAM, NPHS1, NRP1, ORM1, PRSS12, PTPRR, RFPL1/RFPL3, SCG2, SERPINA3, SGCD, SLC2A4, SORCS3, TACSTD2*	12	7

*The genes (red) were up-regulated in HCC mice treated with a high dose of ascorbate and those labelled blue were down-regulated genes.

## Abbreviations

*AGER*: advanced glycosylation end-product specific receptor
Asc: ascorbic acid
*AGEs*: advanced glycation end products
AML: acute myeloid leukaemia
ATO: arsenic trioxide
CC: cholangiocarcinoma
DAG: diacylglycerol
*DGKK*: diacylglycerol kinase kappa
DHE: dihydroethidium
ECAR: extracellular acidification rate
EPCs: endothelial progenitor cells
FBS: foetal bovine serum
FCCP: carbonyl cyanide-4 (trifluoromethoxy) phenylhydrazone
GBM: glioblastoma multiforme
HCC: hepatocellular carcinoma
*HIF-1*: hypoxia-inducible factor
IHC: immunohistochemical
IP: intraperitoneal
IV: intravenous
NAC: N-Acetyl-Cysteine
NSCLC: non-small-cell lung cancer
OCR: oxygen consumption rate
P-AscH^-^: Pharmacologic ascorbate
PBS: phosphate buffered saline
PI: propidium iodide
*RAGE*: Receptor For Advanced Glycosylation End Products
ROS: reactive oxygen species
RT-qPCR: reverse transcription-quantitative polymerase chain reaction
SRC: spare respiratory capacity
*TLT*: *TREM*-like transcript
*VEGF*: vascular endothelial growth factor

## References

[1] Mastrangelo D, Pelosi E, Castelli G, Lo-Coco F, Testa U (2018). Mechanisms of anti-cancer effects of ascorbate: Cytotoxic activity and epigenetic modulation. Blood Cells Mol Dis.

[2] Toth SZ, Lorincz T, Szarka A (2017). Concentration Does Matter: The Beneficial and Potentially Harmful Effects of Ascorbate in Humans and Plants.

[3] Cameron E, Pauling L (1976). Supplemental ascorbate in the supportive treatment of cancer: Prolongation of survival times in terminal human cancer. Proc Natl Acad Sci U S A.

[4] Cameron E, Pauling L (1978). Supplemental ascorbate in the supportive treatment of cancer: reevaluation of prolongation of survival times in terminal human cancer. Proc Natl Acad Sci U S A.

[5] Creagan ET, Moertel CG, O'Fallon JR, Schutt AJ, O'Connell MJ, Rubin J (1979). Failure of high-dose vitamin C (ascorbic acid) therapy to benefit patients with advanced cancer. A controlled trial. N Engl J Med.

[6] Moertel CG, Fleming TR, Creagan ET, Rubin J, O'Connell MJ, Ames MM (1985). High-dose vitamin C versus placebo in the treatment of patients with advanced cancer who have had no prior chemotherapy. A randomized double-blind comparison. N Engl J Med.

[7] Verrax J, Calderon PB (2009). Pharmacologic concentrations of ascorbate are achieved by parenteral administration and exhibit antitumoral effects. Free Radic Biol Med.

[8] Wang C, Lv H, Yang W, Li T, Fang T, Lv G (2017). SVCT-2 determines the sensitivity to ascorbate-induced cell death in cholangiocarcinoma cell lines and patient derived xenografts. Cancer Lett.

[9] Pires AS, Marques CR, Encarnacao JC, Abrantes AM, Mamede AC, Laranjo M (2016). Ascorbic acid and colon cancer: an oxidative stimulus to cell death depending on cell profile. Eur J Cell Biol.

[10] Brandt KE, Falls KC, Schoenfeld JD, Rodman SN, Gu Z, Zhan F (2018). Augmentation of intracellular iron using iron sucrose enhances the toxicity of pharmacological ascorbate in colon cancer cells. Redox Biol.

[11] Schoenfeld JD, Sibenaller ZA, Mapuskar KA, Wagner BA, Cramer-Morales KL, Furqan M (2017). O2(-) and H2O2-Mediated Disruption of Fe Metabolism Causes the Differential Susceptibility of NSCLC and GBM Cancer Cells to Pharmacological Ascorbate. Cancer Cell.

[12] Mustafi S, Sant DW, Liu ZJ, Wang G (2017). Ascorbate induces apoptosis in melanoma cells by suppressing Clusterin expression. Sci Rep.

[13] Ma Y, Chapman J, Levine M, Polireddy K, Drisko J, Chen Q (2014). High-dose parenteral ascorbate enhanced chemosensitivity of ovarian cancer and reduced toxicity of chemotherapy. Sci Transl Med.

[14] Monti DA, Mitchell E, Bazzan AJ, Littman S, Zabrecky G, Yeo CJ (2012). Phase I evaluation of intravenous ascorbic acid in combination with gemcitabine and erlotinib in patients with metastatic pancreatic cancer. PLoS One.

[15] Schoenfeld JD, Sibenaller ZA, Mapuskar KA, Bradley MD, Wagner BA, Buettner GR (2018). Redox active metals and H2O2 mediate the increased efficacy of pharmacological ascorbate in combination with gemcitabine or radiation in pre-clinical sarcoma models. Redox Biol.

[16] Mikirova N, Casciari J, Riordan N, Hunninghake R (2013). Clinical experience with intravenous administration of ascorbic acid: achievable levels in blood for different states of inflammation and disease in cancer patients. J Transl Med.

[17] Duconge J, Miranda-Massari JR, Gonzalez MJ, Jackson JA, Warnock W, Riordan NH (2008). Pharmacokinetics of vitamin C: insights into the oral and intravenous administration of ascorbate. P R Health Sci J.

[18] Padayatty SJ, Sun H, Wang Y, Riordan HD, Hewitt SM, Katz A (2004). Vitamin C pharmacokinetics: implications for oral and intravenous use. Ann Intern Med.

[19] Padayatty SJ, Riordan HD, Hewitt SM, Katz A, Hoffer LJ, Levine M (2006). Intravenously administered vitamin C as cancer therapy: three cases. CMA.

[20] Rouleau L, Antony AN, Bisetto S, Newberg A, Doria C, Levine M (2016). Synergistic effects of ascorbate and sorafenib in hepatocellular carcinoma: New insights into ascorbate cytotoxicity. Free Radic Biol Med.

[21] Fares N, Peron JM (2013). [Epidemiology, natural history, and risk factors of hepatocellular carcinoma]. Rev Prat.

[22] Chen H, Wong CC, Liu D, Go MYY, Wu B, Peng S (2019). APLN promotes hepatocellular carcinoma through activating PI3K/Akt pathway and is a druggable target. Theranostics.

[23] Chedid MF, Kruel CRP, Pinto MA, Grezzana-Filho TJM, Leipnitz I, Kruel CDP (2017). Hepatocellular carcinoma: dignosis and operative management. Arq Bras Cir Dig.

[24] Ma LJ, Feng FL, Dong LQ, Zhang Z, Duan M, Liu LZ (2018). Clinical significance of PD-1/PD-Ls gene amplification and overexpression in patients with hepatocellular carcinoma. Theranostics.

[25] Bomford A, Conlon-Hollingshead C, Munro HN (1981). Adaptive responses of rat tissue isoferritins to iron administration. Changes in subunit synthesis, isoferritin abundance, and capacity for iron storage. The Journal of biological chemistry.

[26] Boser P, Mordashova Y, Maasland M, Trommer I, Lorenz H, Hafner M (2016). Quantification of Hepcidin-related Iron Accumulation in the Rat Liver. Toxicol Pathol.

[27] Gao X, Wei K, Hu B, Xu K, Tang B (2019). Ascorbic acid induced HepG2 cells' apoptosis via intracellular reductive stress. Theranostics.

[28] Parrow NL, Leshin JA, Levine M (2013). Parenteral ascorbate as a cancer therapeutic: a reassessment based on pharmacokinetics. Antioxid Redox Signal.

[29] Sell S (2003). Mouse models to study the interaction of risk factors for human liver cancer. Cancer Res.

[30] Wang J, Chan JY, Fong CC, Tzang CH, Fung KP, Yang M (2009). Transcriptional analysis of doxorubicin-induced cytotoxicity and resistance in human hepatocellular carcinoma cell lines. Liver Int.

[31] Liu TF, Vachharajani V, Millet P, Bharadwaj MS, Molina AJ, McCall CE (2015). Sequential actions of SIRT1-RELB-SIRT3 coordinate nuclear-mitochondrial communication during immunometabolic adaptation to acute inflammation and sepsis. J Biol Chem.

[32] Doskey CM, Buranasudja V, Wagner BA, Wilkes JG, Du J, Cullen JJ (2016). Tumor cells have decreased ability to metabolize H2O2: Implications for pharmacological ascorbate in cancer therapy. Redox biology.

[33] Chen Y, Zhang J, Zhang XY (2015). 2-NBDG as a marker for detecting glucose uptake in reactive astrocytes exposed to oxygen-glucose deprivation *in vitro*. J Mol Neurosci.

[34] Xia Q, Ding T, Zhang G, Li Z, Zeng L, Zhu Y (2018). Circular RNA Expression Profiling Identifies Prostate Cancer- Specific circRNAs in Prostate Cancer. Cell Physiol Biochem.

[35] Yun J, Mullarky E, Lu C, Bosch KN, Kavalier A, Rivera K (2015). Vitamin C selectively kills KRAS and BRAF mutant colorectal cancer cells by targeting GAPDH. Science (New York, NY).

[36] Aguilera O, Munoz-Sagastibelza M, Torrejon B, Borrero-Palacios A, Del Puerto-Nevado L, Martinez-Useros J (2016). Vitamin C uncouples the Warburg metabolic switch in KRAS mutant colon cancer. Oncotarget.

[37] Cho S, Chae JS, Shin H, Shin Y, Song H, Kim Y (2018). Hormetic dose response to L-ascorbic acid as an anti-cancer drug in colorectal cancer cell lines according to SVCT-2 expression. Sci Rep.

[38] Pei Z, Zhang X, Ji C, Liu SM, Wang J (2016). Transcriptomic and functional pathways analysis of ascorbate-induced cytotoxicity and resistance of Burkitt lymphoma. Oncotarget.

[39] Turhal NS, Savas B, Coskun O, Bas E, Karabulut B, Nart D (2015). Prevalence of K-Ras mutations in hepatocellular carcinoma: A Turkish Oncology Group pilot study. Mol Clin Oncol.

[40] Tannapfel A, Sommerer F, Benicke M, Katalinic A, Uhlmann D, Witzigmann H (2003). Mutations of the BRAF gene in cholangiocarcinoma but not in hepatocellular carcinoma. Gut.

[41] Bhaduri A, Ungewickell A, Boxer LD, Lopez-Pajares V, Zarnegar BJ, Khavari PA (2015). Network Analysis Identifies Mitochondrial Regulation of Epidermal Differentiation by MPZL3 and FDXR. Dev Cell.

[42] Lin SC, Karoly ED, Taatjes DJ (2013). The human DeltaNp53 isoform triggers metabolic and gene expression changes that activate mTOR and alter mitochondrial function. Aging cell.

[43] Linster CL, Van Schaftingen E (2007). Vitamin C. Biosynthesis, recycling and degradation in mammals. The FEBS journal.

[44] Hong SW, Lee SH, Moon JH, Hwang JJ, Kim DE, Ko E (2013). SVCT-2 in breast cancer acts as an indicator for L-ascorbate treatment. Oncogene.

[45] Sharma P, Mongan PD (2001). Ascorbate reduces superoxide production and improves mitochondrial respiratory chain function in human fibroblasts with electron transport chain deficiencies. Mitochondrion.

[46] Chen Q, Espey MG, Sun AY, Pooput C, Kirk KL, Krishna MC (2008). Pharmacologic doses of ascorbate act as a prooxidant and decrease growth of aggressive tumor xenografts in mice. Proc Natl Acad Sci U S A.

[47] Vickers AJ, Kuo J, Cassileth BR (2006). Unconventional anticancer agents: a systematic review of clinical trials. J Clin Oncol.

[48] Lv H, Wang C, Fang T, Li T, Lv G, Han Q (2018). Vitamin C preferentially kills cancer stem cells in hepatocellular carcinoma via SVCT-2. NPJ precision oncology.

[49] Schmidt AM, Yan SD, Yan SF, Stern DM (2001). The multiligand receptor RAGE as a progression factor amplifying immune and inflammatory responses. J Clin Invest.

[50] John WG, Lamb EJ (1993). The Maillard or browning reaction in diabetes. Eye (Lond).

[51] Chen J, Jing J, Yu S, Song M, Tan H, Cui B (2016). Advanced glycation endproducts induce apoptosis of endothelial progenitor cells by activating receptor RAGE and NADPH oxidase/JNK signaling axis. Am J Transl Res.

[52] Piras S, Furfaro AL, Domenicotti C, Traverso N, Marinari UM, Pronzato MA (2016). RAGE Expression and ROS Generation in Neurons: Differentiation versus Damage. Oxid Med Cell Longev.

[53] Ohtsu A, Shibutani Y, Seno K, Iwata H, Kuwayama T, Shirasuna K (2017). Advanced glycation end products and lipopolysaccharides stimulate interleukin-6 secretion via the RAGE/TLR4-NF-kappaB-ROS pathways and resveratrol attenuates these inflammatory responses in mouse macrophages. Exp Ther Med.

[54] Sakane F, Mizuno S, Komenoi S (2016). Diacylglycerol Kinases as Emerging Potential Drug Targets for a Variety of Diseases: An Update. Front Cell Dev Biol.

[55] van der Zanden LF, van Rooij IA, Feitz WF, Knight J, Donders AR, Renkema KY (2011). Common variants in DGKK are strongly associated with risk of hypospadias. Nat Genet.

[56] Banan A, Fields JZ, Zhang Y, Keshavarzian A (2001). Phospholipase C-gamma inhibition prevents EGF protection of intestinal cytoskeleton and barrier against oxidants. Am J Physiol Gastrointest Liver Physiol.

[57] Franklin RA, Atherfold PA, McCubrey JA (2000). Calcium-induced ERK activation in human T lymphocytes occurs via p56(Lck) and CaM-kinase. Mol Immunol.

[58] Dann SG, Golas J, Miranda M, Shi C, Wu J, Jin G (2014). p120 catenin is a key effector of a Ras-PKCvarepsilon oncogenic signaling axis. Oncogene.

[59] Ali MA, Eid R, Hanafi MY (2018). Vitamin C and E chronic supplementation differentially affect hepatic insulin signaling in rats. Life Sci.

[60] Carcamo JM, Borquez-Ojeda O, Golde DW (2002). Vitamin C inhibits granulocyte macrophage-colony-stimulating factor-induced signaling pathways. Blood.

[61] Phillips DC, Allen K, Griffiths HR (2002). Synthetic ceramides induce growth arrest or apoptosis by altering cellular redox status. Arch Biochem Biophys.

[62] Zhuang H, Qiang Z, Shao X, Wang H, Dang Y, Wang Z (2019). Integration of metabolomics and expression of enolase-phosphatase 1 links to hepatocellular carcinoma progression. Theranostics.

