# Supplementary material for: Hepatomas are exquisitely sensitive to pharmacologic ascorbate (P-AscH-)
Source: Theranostics. 2019 Oct 18;9(26):8109–26. doi: 10.7150/thno.35378 (PMC6857065; doi:10.7150/thno.35378)

Supplementary information:

Hepatomas are exquisitely sensitive to pharmacologic ascorbate (P-AscH$^-$)

Xuan Zhang[1,#], Tiefu Liu[1,#], Zehuan Li[2,#], Yanling Feng[1], Christopher Corpe[3], Shanshan Liu[1], Jingpu Zhang[1], Xiaomeng He[1], Feng Liu[1], Li Xu[1], Longqiang Shen[1], Shun Li[1], Qianlin Xia[4], Xiuhua Peng[1], Xiaohui Zhou[1], Weiping Chen[5], Xiaoyan Zhang[1], Jianqing Xu[1], Jin Wang[1,*]

[1]Shanghai Public Health Clinical Center, Fudan University, 2901 Caolang Road, Jinshan District, Shanghai 201508, China; [2]Department of General Surgery, Zhongshan Hospital, Fudan University, 200032, Shanghai, China; [3]King's College London, London, Nutritional Science Department, 150 Stamford street, waterloo, London, SE19NH, United Kingdom; [4]Department of Laboratory Medicine, Shanghai Jiao Tong University Affiliated Sixth People's Hospital, Shanghai, China; [5]Genomics Core, National Institute of Diabetes and Digestive and Kidney Diseases, National Institutes of Health, Bethesda, MD 20892, USA.

[#] These authors contributed equally to this work

[*]**Corresponding Author:**
Jin Wang, Ph.D.

Scientific Research Center,

Shanghai Public Health Clinical Center,

Fudan University,

2901 Caolang Road, Jinshan District, Shanghai 201508, China;

Ph: 86-21-57036495; Fax: 86-21-57247094

Email: wjincityu@yahoo.com

1. Supplemental Tables.

1.1. Table S1. Sequences of primers of differentially expressed genes for real-time qPCR.

**Name of genes**	**Sequences of Primers (5'-3')**	
	Forward	Reverse
CDK4	ATGGACGTCTGTGCCACATC	CACGGGTGTAAGTGCCATCT
CDK6	ACAGTGTCACGAACAGACAG	CAAGACTTCGGGTGCTCTGTA
c-Myc	CCTTCTCTCCGTCCTCGGAT	TGCTGATGTGTGGAGACGTG
Casp3	ATGTCGATGCAGCAAACCTC	CCAGTTCTGTACCACGGCAG
AGER	GCCACTGGTGCTGAAGTGTA	CACGGACTCGGTAGTTGGAC
DGKK	TGAAGGAAGGACCTATGCTGA	ATGTCTTTCCGGTTGGGTGC
ASB2	ACCTGGACTGTCTCCTGTCA	TTGGATTCCACCTTGGCTCC
TCP10L2	TGGATGCCTTGAGGAAGCAG	AGTCGCACTCTGACTCTTGC
Lnc-ALCAM-3	AGACCATGCTCCTTCCACAT	TAAAGCTGCCAACAACTCCG
lnc-TGFBR2-1	GGCATGGACAGATCTCATTGG	GGGAGAGTCTTGAGCTTGGT
18S	GTAACCCGTTGAACCCCATT	CCATCCAATCGGTAGTAGCG

1.2. Table S2. 192 differentially expressed genes were only in High dose of ascorbate treated HCC.

Probeset ID	GeneName	Fold-Change(H vs. N)	p-value(H vs. N)
NM_001206954	AGER	10.14	0.049
ENST00000522882	ENST00000522882	9.52	0.042
lnc-DLEU1-1:1	lnc-DLEU1-1	9.44	0.029
lnc-ALCAM-3:1	lnc-ALCAM-3	5.45	0.044
lnc-TGFBR2-1:1	lnc-TGFBR2-1	4.47	0.033
NM_001242473	C16orf97	4.18	0.023
ENST00000593792	ENST00000593792	3.85	0.040
NM_001013742	DGKK	3.60	0.030
NM_014858	TMCC2	3.59	0.038
TCONS_l2_00009686	XLOC_l2_005187	3.59	0.029
NM_000815	GABRD	3.58	0.031
NM_001322	CST2	3.54	0.023
lnc-BTF3L4-2:1	lnc-BTF3L4-2	3.48	0.032
XR_246983	LOC101927345	3.42	0.021
ENST00000544271	BAD	3.42	0.032
NM_001452	FOXF2	3.37	0.038
NM_152450	FAM81A	3.33	0.049
NR_125797	ZFPM2-AS1	3.30	0.030
ENST00000564702	ENST00000564702	3.29	0.039
NR_040093	STARD4-AS1	3.24	0.036
lnc-RNF38-2:1	lnc-RNF38-2	3.23	0.024
NM_002023	FMOD	3.20	0.028
NR_003191	GGTA1P	3.17	0.044
ENST00000541749	LOC101929027	3.08	0.036
AK022058	AK022058	3.02	0.043
NM_001024457	RGPD1	2.97	0.041
XR_424822	LOC101929494	2.94	0.036
lnc-KIF20B-1:3	lnc-KIF20B-1	2.93	0.026
NM_178826	ANO4	2.83	0.021
NR_110565	MCM3AP-AS1	2.81	0.040
NM_173662	RNF175	2.79	0.041
NR_046575	MAGI1-AS1	2.78	0.036
NM_001184746	PAFAH1B2	2.78	0.031
NM_004982	KCNJ8	2.77	0.044
NR_003072	SNORD91A	2.74	0.049
lnc-C1orf86-1:2	lnc-C1orf86-1	2.73	0.044
NM_021992	TMSB15A	2.69	0.023
NM_000541	SAG	2.66	0.047
XR_424301	LOC101929584	2.66	0.048

NM_030949	PPP1R14C	2.66	0.046
NM_001190972	C8orf88	2.65	0.018
NR_028371	FAS-AS1	2.64	0.025
lnc-TRAF5-4:1	lnc-TRAF5-4	2.61	0.033
NM_172006	WFDC10B	2.59	0.029
NR_003141	SNHG4	2.58	0.046
lnc-AC092031.1-1:11	lnc-AC092031.1-1	2.54	0.037
NM_001406	EFNB3	2.51	0.042
ENST00000370434	ABCC2	2.51	0.041
lnc-RPS6KA3-1:1	lnc-RPS6KA3-1	2.49	0.042
NM_001001953	OR10G9	2.48	0.042
AB007978	KIAA0509	2.38	0.023
NR_036536	SNHG4	2.32	0.024
NR_024014	PP14571	2.32	0.049
NM_032598	SPATA22	2.32	0.031
lnc-TAF15-1:1	lnc-TAF15-1	2.28	0.049
NM_005810	KLRG1	2.28	0.034
NM_004750	CRLF1	2.25	0.045
lnc-AC007557.1.1-3:2	lnc-AC007557.1.1-3	2.25	0.043
NR_033791	DOC2GP	2.24	0.031
NM_033119	NKD1	2.23	0.037
NM_001005610	EDA	2.20	0.047
ENST00000374186	DHDDS	2.19	0.041
lnc-PPT1-1:1	lnc-PPT1-1	2.18	0.044
lnc-TRMT11-1:1	lnc-TRMT11-1	2.16	0.028
lnc-BTBD10-3:10	lnc-BTBD10-3	2.12	0.048
NR_026704	VTRNA1-2	2.12	0.035
NM_175630	DNMT3A	2.11	0.021
ENST00000617024	SPTSSB	2.11	0.032
NR_038287	TEX26-AS1	2.11	0.039
NM_198501	SMTNL2	2.10	0.045
lnc-AC092031.1-1:8	lnc-AC092031.1-1	2.09	0.040
NM_006209	ENPP2	2.08	0.036
BI056255	BI056255	2.07	0.045
lnc-NSMAF-1:1	lnc-NSMAF-1	2.05	0.021
NM_003619	PRSS12	2.04	0.048
ENST00000392645	ATXN2	2.04	0.045
NM_203486	DLL3	2.03	0.028
ENST00000605570	ENST00000605570	2.01	0.042
NM_001005463	EBF3	2.01	0.034
THC2682558	THC2682558	2.01	0.030
NM_024534	ERVMER34-1	2.00	0.043
NR_036446	ACTG1P17	-2.01	0.049
lnc-CCDC40-1:1	lnc-CCDC40-1	-2.01	0.045

lnc-MINA-3:1	lnc-MINA-3	-2.02	0.027
lnc-KANK4-1:1	lnc-KANK4-1	-2.03	0.031
THC2701566	THC2701566	-2.06	0.030
ENST00000427341	ENST00000427341	-2.07	0.043
NM_001110199	SRRM3	-2.07	0.038
NM_012114	CASP14	-2.07	0.035
NM_021026	RFPL1	-2.10	0.037
NM_033317	DMKN	-2.10	0.049
lnc-RGL4-4:2	lnc-RGL4-4	-2.11	0.039
lnc-TELO2-3:1	lnc-TELO2-3	-2.13	0.049
ENST00000421673	MUC6	-2.13	0.046
lnc-NDE1-3:1	lnc-NDE1-3	-2.15	0.046
NM_003027	SH3GL3	-2.15	0.022
lnc-RP11-150O12.3.1-3:1	lnc-RP11-150O12.3.1-3	-2.15	0.043
NM_001002294	FMO3	-2.16	0.039
ENST00000538329	ENST00000538329	-2.16	0.044
NR_001296	PRSS3P2	-2.18	0.037
NM_000478	ALPL	-2.20	0.033
NR_030732	WFDC21P	-2.22	0.043
lnc-NAA35-1:2	lnc-NAA35-1	-2.22	0.042
lnc-SALL3-2:1	lnc-SALL3-2	-2.23	0.048
ENST00000528496	LOC101928823	-2.23	0.036
ENST00000517994	ENST00000517994	-2.23	0.033
lnc-ID4-1:1	lnc-ID4-1	-2.24	0.039
NM_001171832	DEFB121	-2.24	0.023
THC2674454	THC2674454	-2.25	0.033
A_33_P3351791	A_33_P3351791	-2.25	0.045
lnc-LRP11-1:1	lnc-LRP11-1	-2.25	0.018
NM_001650	AQP4	-2.27	0.045
NM_001017915	INPP5D	-2.28	0.025
lnc-TGFBRAP1-11:1	lnc-TGFBRAP1-11	-2.29	0.032
NR_040094	LINC01193	-2.30	0.045
CU688821	CU688821	-2.31	0.044
NM_018995	MOV10L1	-2.32	0.045
NM_001003954	ANXA13	-2.36	0.045
TCONS_l2_00014098	XLOC_l2_007656	-2.37	0.042
NM_014725	STARD8	-2.37	0.047
ENST00000450729	ENST00000450729	-2.37	0.024
lnc-MFAP4-4:1	lnc-MFAP4-4	-2.39	0.045
NM_032045	KREMEN1	-2.39	0.030
TCONS_l2_00009528	XLOC_l2_005020	-2.40	0.043
lnc-C5orf17-5:1	lnc-C5orf17-5	-2.41	0.047
XR_433481	LOC101927943	-2.42	0.028
NM_001105581	LRRC30	-2.42	0.032

lnc-EBF3-1:1	lnc-EBF3-1	-2.43	0.048
lnc-ITGA7-1:1	lnc-ITGA7-1	-2.44	0.043
ENST00000517369	ENST00000517369	-2.47	0.042
lnc-IDS-5:1	lnc-IDS-5	-2.50	0.038
NM_017986	SLC52A1	-2.52	0.023
AA378382	SNORA12	-2.52	0.037
NR_024507	LINC00598	-2.53	0.020
lnc-NME4-1:2	lnc-NME4-1	-2.53	0.033
NM_000751	CHRND	-2.54	0.040
NM_001307	CLDN7	-2.54	0.029
lnc-ITPR2-2:3	lnc-ITPR2-2	-2.54	0.041
NM_000337	SGCD	-2.56	0.039
lnc-ACOT12-2:1	lnc-ACOT12-2	-2.56	0.042
NR_003062	SPRR2C	-2.58	0.034
lnc-LENG9-2:1	lnc-LENG9-2	-2.59	0.043
lnc-SGCG-5:1	lnc-SGCG-5	-2.59	0.043
NM_001001850	STX19	-2.60	0.049
lnc-RNF152-1:1	lnc-RNF152-1	-2.64	0.022
NM_004205	USP2	-2.65	0.048
AK096685	AK096685	-2.69	0.032
lnc-TRUB2-2:1	lnc-TRUB2-2	-2.70	0.045
A_21_P0014374	A_21_P0014374	-2.70	0.031
AK127488	LOC100130429	-2.71	0.048
NM_032648	FAM167B	-2.73	0.039
A_33_P3368445	A_33_P3368445	-2.74	0.030
NM_020630	RET	-2.74	0.046
lnc-MTERFD3-2:1	lnc-MTERFD3-2	-2.82	0.023
ENST00000572608	LOC102723363	-2.83	0.048
ENST00000510838	ENST00000510838	-2.84	0.049
NM_001078	VCAM1	-2.84	0.046
NR_122070	LOC729083	-2.85	0.038
lnc-RXFP4-1:1	lnc-RXFP4-1	-2.89	0.030
NM_032411	C2orf40	-2.91	0.044
NR_033312	BDNF-AS	-2.92	0.035
ENST00000578788	ENST00000578788	-2.94	0.042
A_33_P3417547	A_33_P3417547	-2.95	0.045
XR_241421	LOC101928662	-2.96	0.038
lnc-LRRTM2-1:1	lnc-LRRTM2-1	-2.99	0.045
NR_120410	LMO7-AS1	-3.04	0.035
NM_024889	PLEKHS1	-3.09	0.050
A_33_P3351166	A_33_P3351166	-3.09	0.046
CA436475	SNAR-E	-3.14	0.021
lnc-CDYL2-6:5	lnc-CDYL2-6	-3.15	0.034
ENST00000585853	PSMG2	-3.18	0.048

TCONS_l2_00008966	XLOC_l2_004857	-3.19	0.039
NM_001038	SCNN1A	-3.27	0.035
NM_021175	HAMP	-3.30	0.026
ENST00000421132	ENST00000421132	-3.34	0.032
NR_026836	TRHDE-AS1	-3.40	0.034
lnc-TMTC4-1:1	lnc-TMTC4-1	-3.54	0.043
lnc-EN1-1:1	lnc-EN1-1	-3.61	0.025
lnc-EEA1-1:2	lnc-EEA1-1	-3.93	0.034
lnc-ADAMTS17-1:6	lnc-ADAMTS17-1	-3.95	0.027
THC2570812	THC2570812	-3.97	0.045
NM_005755	EBI3	-4.01	0.035
lnc-FAM27D1.1-3:1	lnc-FAM27D1.1-3	-4.07	0.020
NM_016150	ASB2	-4.09	0.029
NM_001145121	TCP10L2	-4.16	0.040
ENST00000433724	ENST00000433724	-4.21	0.035
A_33_P3809328	A_33_P3809328	-4.41	0.045
NM_001145641	SRRM5	-4.71	0.028
lnc-GLB1L2-1:11	lnc-GLB1L2-1	-4.80	0.045
lnc-WNT7A-1:3	lnc-WNT7A-1	-5.07	0.037
THC2715267	THC2715267	-5.29	0.032
ENST00000426418	ENST00000426418	-9.16	0.019

1.3. Table S3. Top five pathways of 192 DEGs in HCC mice treated with high dose of ascorbate by IPA analysis.

Ingenuity Canonical Pathways	-log(p-value)	Ratio	Molecules
Insulin Receptor Signaling	2.92E+00	2.84E-02	SCNN1A, PPP1R14C, BAD, INPP5D
Dolichol and Dolichyl Phosphate Biosynthesis	2.18E+00	5.00E-01	DHDDS
UDP-N-acetyl-D-glucosamine Biosynthesis II	1.71E+00	1.67E-01	FMO3
Ceramide Biosynthesis	1.64E+00	1.43E-01	SPTSSB
IL-3 Signaling	1.51E+00	2.41E-02	BAD, INPP5D

2. Supplemental Figures:

2.1. Figure S1. Soft-agar assay of Huh-7 and LO2 cells treated with ascorbate for 14-21 days. A) Huh-7 cells; B) LO2 cells.

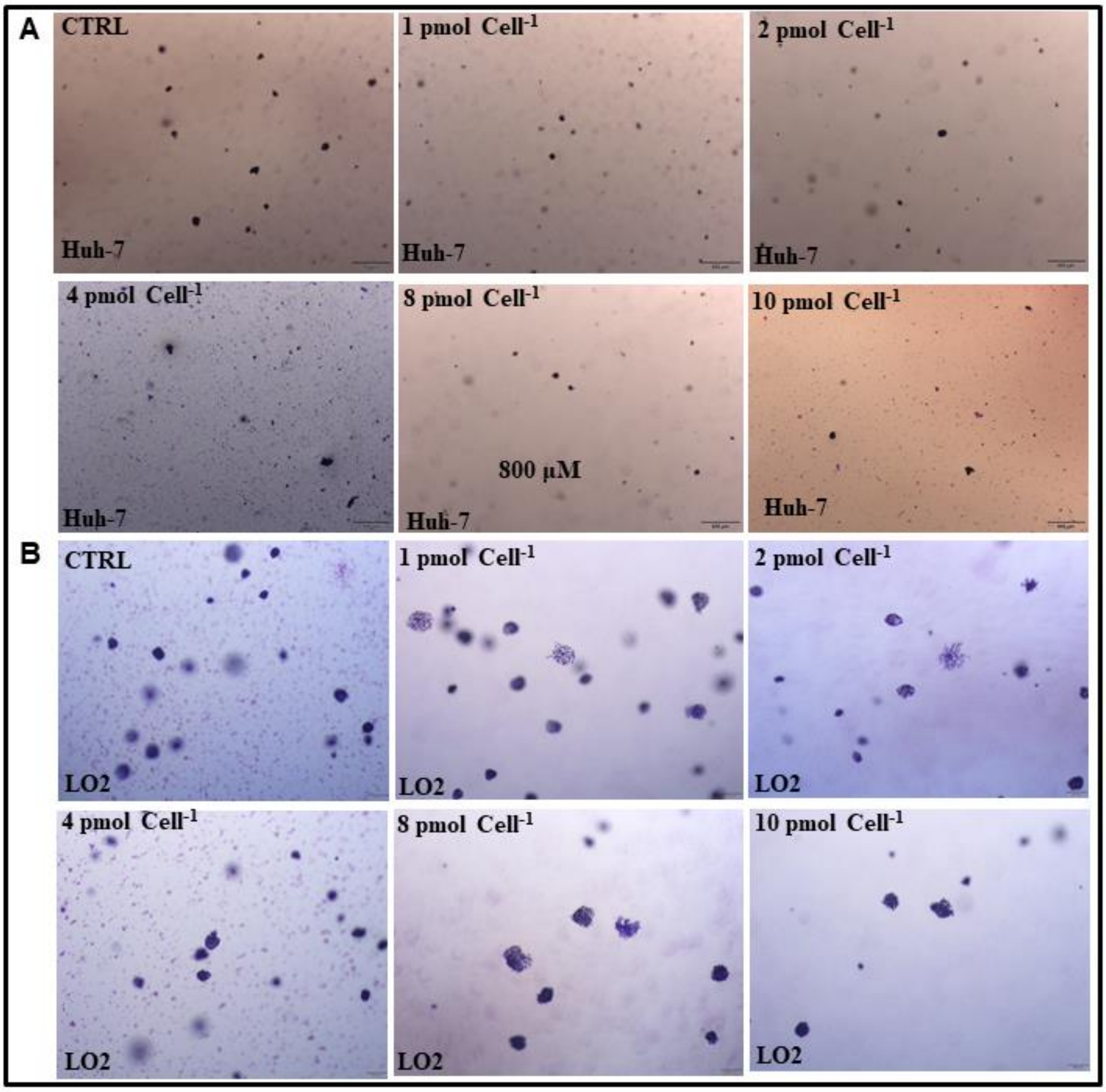

2.2. Figure S2. Kyoto Encyclopedia of Genes and Genomes (KEGG) analysis of these differentially expressed mRNAs was performed to determine the top 30 pathways of the differential mRNAs in HCC tumour tissue from mice treated with IP injection of ascorbate at 2.0 g/kg/3 days (A) and 4.0 g/kg/3 days (B) compared with expression in their controls.

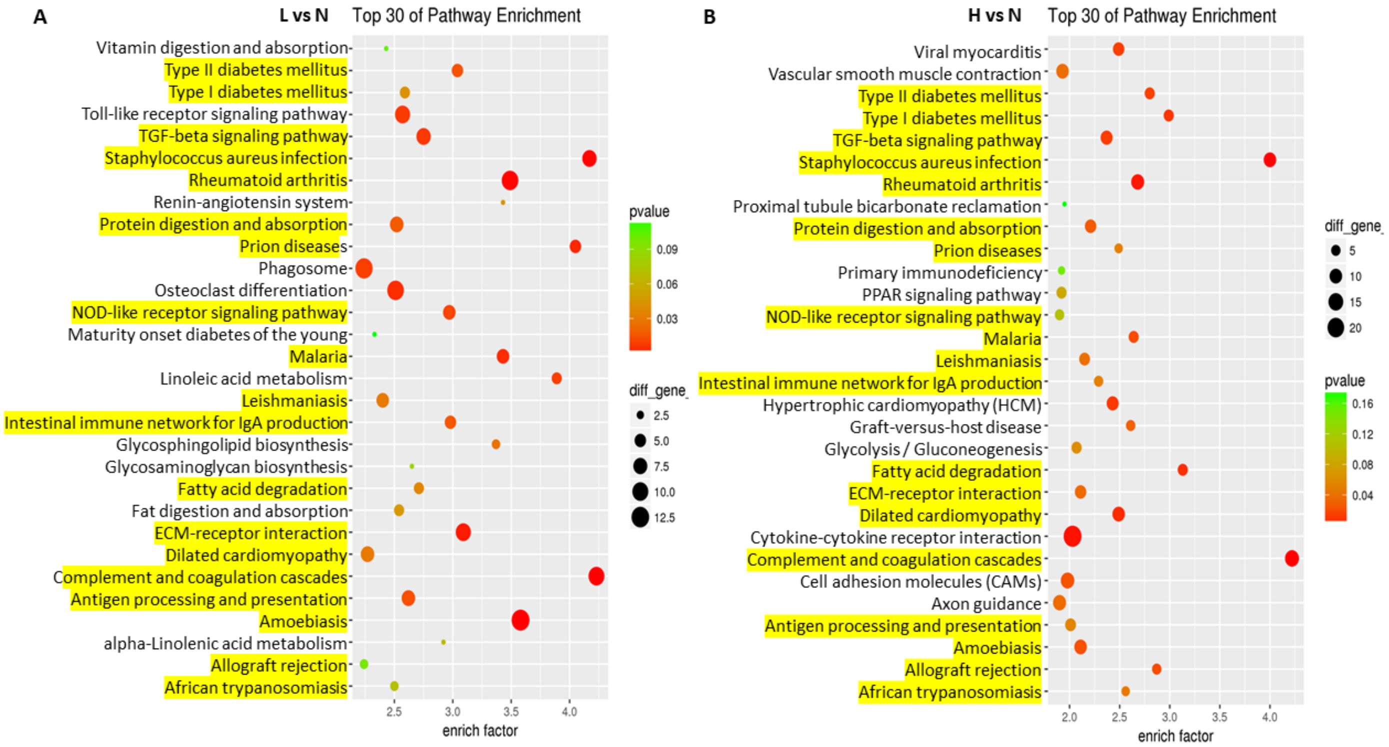

2.3. Figure S3. PET/CT scan images of the patients of middle age woman with massive primary cancer of the liver (hepatocellular carcinoma) with 4treatments of HiCLoChemo (B), compared with her PET/CT scan done before the start of treatment (A).

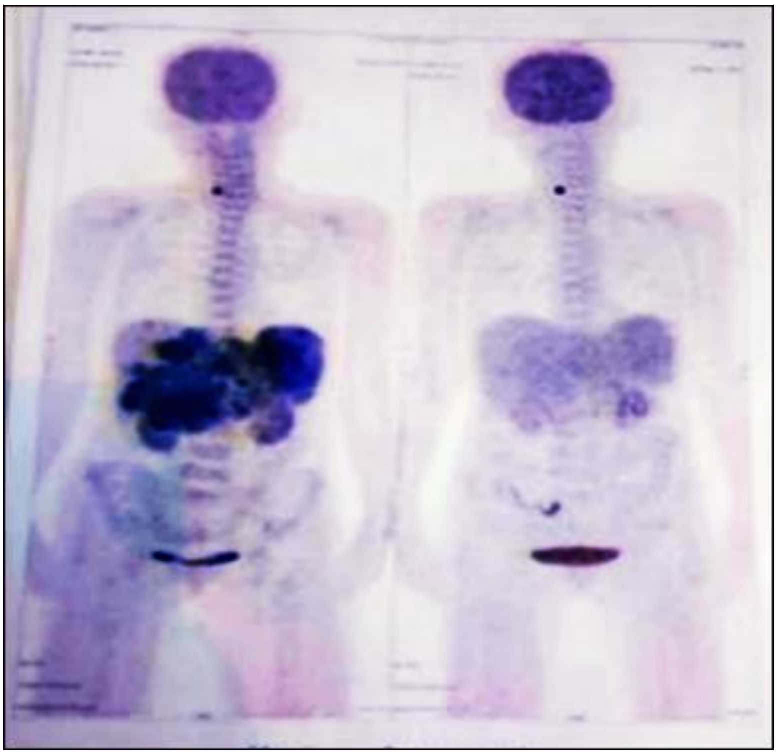

A B

2.4. Figure S4. The description of the generation of the ascorbate in animals and plants which can synthesize ascorbate through a sequence of enzyme-driven steps involving in the converting monosaccharides to ascorbate.

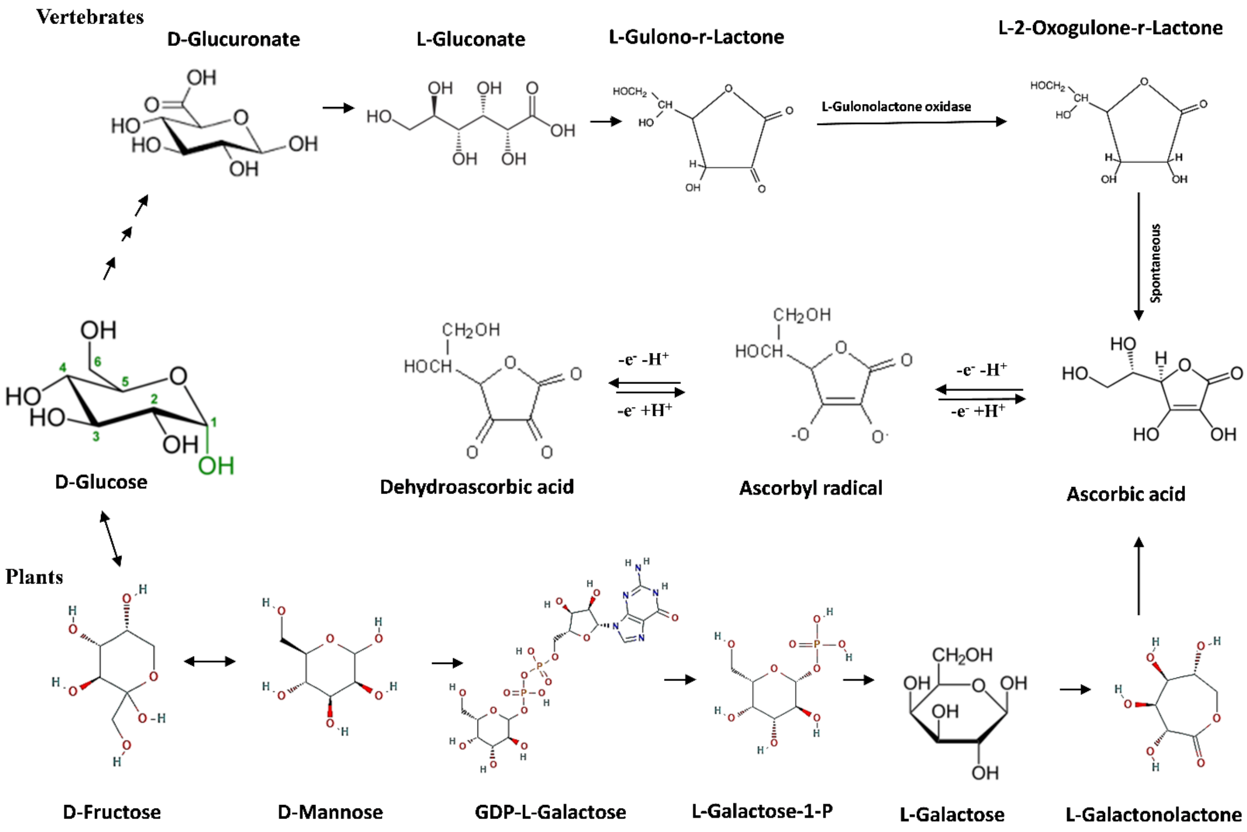

Supplement: Supplementary file 1 — Supplementary figures and tables. [file thnov09p8109s1.pdf]
